# DIGE Proteome Analysis Reveals Suitability of Ischemic Cardiac *In Vitro* Model for Studying Cellular Response to Acute Ischemia and Regeneration

**DOI:** 10.1371/journal.pone.0031669

**Published:** 2012-02-22

**Authors:** Sina Haas, Heinz-Georg Jahnke, Nora Moerbt, Martin von Bergen, Seyedhossein Aharinejad, Olena Andrukhova, Andrea A. Robitzki

**Affiliations:** 1 Division of Molecular Biological-Biochemical Processing Technology, Center for Biotechnology and Biomedicine, Universität Leipzig, Leipzig, Germany; 2 Department of Proteomics, Helmholtz Centre for Environmental Research, Leipzig, Germany; 3 Department of Metabolomics, Helmholtz Centre for Environmental Research, Leipzig, Germany; 4 Department of Cardiac Surgery, Center of Anatomy and Cell Biology, Medical University of Vienna, Vienna, Austria; 5 Department for Cardiovascular Research, Center of Anatomy and Cell Biology, Medical University of Vienna, Vienna, Austria; 6 Department for Biomedical Sciences, Institute of Pathophysiology, University of Veterinary Medicine, Vienna, Austria; Hemocentro de Ribeirão Preto - HC-FMRP-USP., Brazil

## Abstract

Proteomic analysis of myocardial tissue from patient population is suited to yield insights into cellular and molecular mechanisms taking place in cardiovascular diseases. However, it has been limited by small sized biopsies and complicated by high variances between patients. Therefore, there is a high demand for suitable model systems with the capability to simulate ischemic and cardiotoxic effects *in vitro*, under defined conditions. In this context, we established an *in vitro* ischemia/reperfusion cardiac disease model based on the contractile HL-1 cell line. To identify pathways involved in the cellular alterations induced by ischemia and thereby defining disease-specific biomarkers and potential target structures for new drug candidates we used fluorescence 2D-difference gel electrophoresis. By comparing spot density changes in ischemic and reperfusion samples we detected several protein spots that were differentially abundant. Using MALDI-TOF/TOF-MS and ESI-MS the proteins were identified and subsequently grouped by functionality. Most prominent were changes in apoptosis signalling, cell structure and energy-metabolism. Alterations were confirmed by analysis of human biopsies from patients with ischemic cardiomyopathy.

With the establishment of our *in vitro* disease model for ischemia injury target identification via proteomic research becomes independent from rare human material and will create new possibilities in cardiac research.

## Introduction

Cardiac diseases and myocardial dysfunctions following ischemia are the leading cause of mortality in western industrialized countries. Ischemia and reperfusion injury, resulting from clinical setting of coronary revascularization in acute myocardial infarction, bypass surgery and heart transplantation is a demanding issue. Many dysfunctions and defects have been described to be responsible for the occurrence of ischemic injury; degeneration of cytoskeleton [1], disturbances in calcium homeostasis [2], generation of reactive oxygen species [3], loss of high-energy phosphates [4] and the occurrence of suicidal cell death [5], [6] may play a crucial role in pathogenesis. Treatment of myocardial infarction to restore blood flow to the ischemic region by thrombolysis or coronary artery bypass surgery leads either to hypoxic myocardial tissue, where necrotic and wound-healing process is initiated [7] and contractile function is lost; or blood flow through the myocardium is re-established in time and tissue may regain its function, but may also experience additional damage due to the reperfusion process itself by generation of reactive oxygen species [8], [9].

Proteomic studies of human disease derived pathological altered tissue can provide new insights into the molecular mechanisms that underlie the responses to ischemia and reperfusion injury. These findings are important for drug development and could lead to new approaches for novel therapeutic strategies. Two-dimensional difference in gel electrophoresis (2D-DIGE) is a potential tool for target identification, because fluorescence labelling offers the possibility to separate and compare healthy and diseased samples on one gel and analyse differences in a quantitative manner by omitting gel to gel variances and a reliable quantification [10].

Although human biopsy samples are the most suitable source of material for identification of biomarkers and potential drug targets in proteomic approaches, the size of individual samples is limited. Another limitative aspect is the availability of well characterized material that is normalized for the following criteria: disease state, tissue heterogeneity, genetic variability, gender specificity, medical history and therapeutic interventions [11]. Hence a model system is needed, where pathological effects of ischemia and ischemia-reperfusion could be properly simulated. To date a couple of standardized animal models are used to simulate myocardial infarction and ischemia-reperfusion *in vivo* and changes in their myocardial proteomes were identified by 2D-gel electrophoresis [12]–[16]. Here we describe the use of an *in vitro* model to investigate effects of myocardial ischemia and ischemia-reperfusion under controlled conditions. The mouse derived HL-1 cardiomyocyte cell line is the only cell line that can be passaged indefinitely in culture while maintaining their contractile activity and phenotypic characteristics of the adult cardiomyocyte *in vitro*. HL-1 cells were intensively studied and characterized in terms of cardiac morphological, biochemical and electrophysiological properties. They show a high similarity in cardiac marker expression of cardiac-specific myosin and muscle-specific desmin intermediate filaments, syncytium formation as well as in morphological and electrophysiological characteristics compared to *in vivo* cardiomyocytes [17]. Because of these unique features, HL-1 cells are the mean of choice for the *in vitro* simulation of heart infarct, resulting in numerous studies that are described in the literature including cultivation under hypoxic conditions [18], nutrient deficiency [19] or oxidative stress [20].

The goal of the present study was to establish a HL-1 cardiomyocyte *in vitro* pathology model that recapitulates major aspects of ischemia and ischemia-reperfusion injury and identify changes in cardiac proteome as well as compare the results with proteome data obtained from human biopsies to validate our ischemic in *vitro* model system. With the help of these findings new potential targets for drug discovery and drug development in cardiac diseases could be identified.

## Results

### Establishment of an ischemic *in vitro* cell culture model

**Figure 1:**
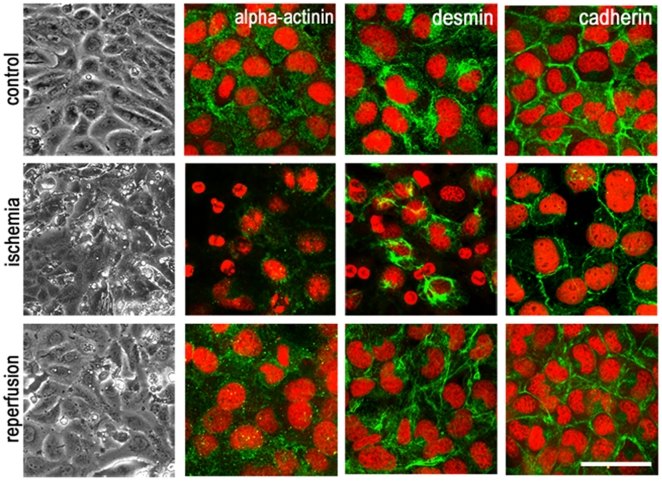
Analysis of ischemia induced changes in the cellular structure of HL-1 cardiomyocytes. While control and reperfused cells show a vital cell layer consists of contractile HL-1 cardiomyocytes and an intact cytoskeleton structure, 8 h after ischemia-induction cells die off and detached from the layer-assembly, indicated by reflecting microscopic structures. Furthermore contractility of HL-1 cardiomyocytes was stopped. Immunocytochemical staining of this phase offered a degradation of the cytoskeleton filaments alpha-actinin, desmin and cadherin, which were labelled with Cy™-antibodies (green). Nuclei were stained using Sytox®-orange. Scale bar microscopic images = 25 µm, fluorescence images = 50 µm.

With the objective to develop an *in vitro* culture model for cardiac ischemia, we established a disease model of using the spontaneous contractile cardiac HL-1 cell line, were pathological effects of ischemia and ischemia-reperfusion injury can be simulated. For induction of ischemia *in vitro*, the standard used Claycomb-medium was replaced by a nutrient deficiency medium additionally contained 25 µM of hydrogen peroxide to enhance the oxidative stress [21]. Microscopic investigations of ischemic cell layers show a degeneration of the connected cell structure with increasing incubation time in ischemic buffer (Fig. 1). Single cells switched over to a stage of early cell death and detached from the monolayer within the first hours of ischemia. Furthermore, decreasing of contraction rate and power could be observed in ischemic HL-1 cultures using electrophysiological field potential measurement (Fig. S1), whereas control cells showed no interferences in monolayer structure and contractile behaviour. To investigate effects caused by ischemia-reperfusion, a revitalization period was initiated by medium exchange with standard Claycomb-medium and cells retained for 16 h. After revitalization time cell culture display a regeneration of aggrieved cells by the rebuild of a complex monolayer. In addition, we observed a revival of cardiac contraction.

### 2D DIGE analyses of ischemic and ischemia-reperfused *in vitro* cultures

**Figure 2:**
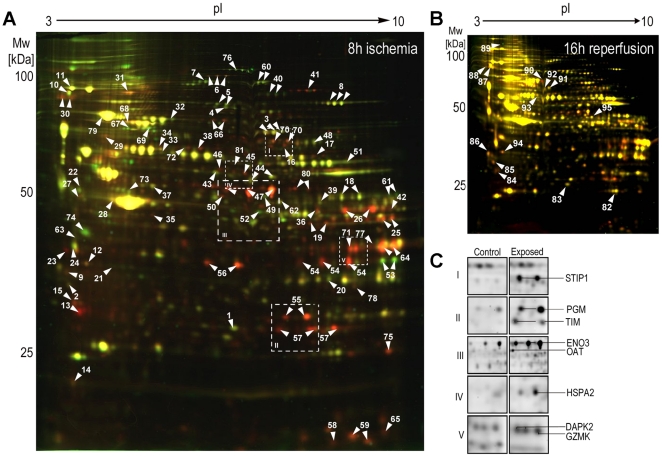
Proteome changes in HL-1 cardiomyocyte cell line with induction of ischemic conditions and after reperfusion. In total 360 µg of cytosolic proteins of ischemia-induced HL-1 cells (A) and reperfused HL-1 cells (B) were separated by DIGE-2-DE (18 cm IPG stripes 3–10 non-linear, 10% acrylamide/bisacrylamide). C) Gel image regions of differentially expressed proteins following ischemia induction. Abbreviations are gene names. The details of identifications are shown in Table S1 and Table S2.

Changes in protein expression profile after 8 h of induced ischemia and after 16 h of ischemia-reperfusion were analysed using the DIGE 2-DE approaches (Fig. 2). We detected 1435 protein spots in the ischemic HL-1 proteome by 2-DE and quantified the expression of ischemic HL-1 cells with p*I* between 3 and 10 using Delta 2D software, version 3.6, yielding 644 spots displaying a difference in protein expression stronger than 2-fold (*p*<0.05). In general, we detected more repressed (441) than induced protein spots (203) after 8 h incubation in ischemic buffer (Fig. 2A). Comparison between ischemic samples and controls showed a clear clustered Heatmap according to the experiments and a high similarity between the three independent replicates (Fig. S2). By using an unsupervised approach comprising all spots the gels were clustered into the correct groups. Changes in expression profile after revitalization in comparison to the control were reduced to a minimum, indicating a regeneration of HL-1 cells during re-incubation in Claycomb-medium and an approximation to the control state (Fig. 2B). Alike expression profile of 8 h samples, we detected more repressed than induced protein spots in revitalized HL-1 cardiomyocytes. By tryptic digest and MS analysis of the peptides in order to create a proteome map of HL-1 cells, we identified 81 differentially expressed proteins from ischemic HL-1 cells (Table S2) and 14 protein spots, which were differentially expressed after reperfusion-time using MALDI-MS (Table S3). In detail, identified proteins are involved in cell death signalling (death associated protein kinase 2, granzyme K), oxidative stress regulation (stress induced phosphoproteine 1), protein quality control (HSPA2), cell structure organisation or metabolism related proteins (Fig. 2C, Table 1).

**Table 1:** Identified proteins from differentially abundant spots of differentially expressed proteins following ischemia/reperfusion in HL-1 cardiomyocytes.

ID^a^	Protein involved in process	Accession^b^	Fold change^c^			
			gel1	gel2	gel3	fusion
**Cell death signaling/apoptosis**						
12	Annexin A5	P48036	1.972	0.436	0.741	1.049
6	Vinculin	Q64727	1.982	1.547	2.049	1.848*
78	Granzyme K	Q9R0K0	7.800	-	7.254	7.527
71	Death associated protein kinase 2	Q9UIK4	-	7.949	7.543	7.746***
2	Tyrosin-3/tryptophan-5-monooxygenase	P62259	0.380	0.173	0.321	0.291
13	Protein kinase C inhibitor protein 1, 14-3-3-zeta	P61101	3.408	3.675	2.810	3.298
49	Eukaryotic translation initiation factor 4A	Q91VC3	0.510	0.533	0.769	0.604
58	Nucleoside diphosphate kinase 2	Q01768	4.706	5.454	6.271	5.477
61	Serpin h1 (Ser/Cys Proteinase Inhibitor) (HSP47)	Q5U4D0	0.098	0.522	0.170	0.263
67	Lamin B1	Q61791	0.388	0.556	0.545	0.496**
63	Nucleophosmin 1	Q61937	0.320	0.567	0.459	0.449**
**Oxidative stress**						
1	Peroxyredoxin 3	P30048	1.267	1.184	-	1.227
44	Aldehyde dehydrogenase 2, mitochondrial	P47738	0.399	0.615	0.545	0.519*
70	Stress induced phosphoprotein	Q60864	2.108	2.147	-	2.158*
31	Valosin containing protein	Q01853	2.900	1.606	1.632	1.956**
46	Selenuim binding protein	P17563	5.898	2.061	3.165	3.708**
75	NADH-dehydrogenase (ubiquinone) 1-beta	Q9CR21	3.121	2.230	1.974	2.442
55	Phosphoglyceratmutase	Q9DBJ1	1.823	1.955	1.581	1.786
						
**Protein quality control**						
10	Heat shock protein 90 - beta (HSP 84)	P11499	0.342	0.051	0.037	0.143
11	Heat shock protein 90 - alpha (HSP 86)	P46633	0.220	-	0.037	0.129
63	Nucleophosmin 1	Q61937	0.320	0.567	0.459	0.449**
30	Heat shock protein 1	P11499	1.532	1.996	1.752	1.760
32	Heat shock protein 9	P38647	0.377	0.223	0.241	0.280
34	Heat shock protein 65	P63038	4.770	7.147	4.229	5.382***
45	Chaperonin 2 (beta) (HSPA2)	P80314	5.825	2.649	3.413	3.962**
61	Serpin h1 (Ser/Cys proteinase inhibitor) (HSP47)	Q5U4D0	0.098	0.522	0.170	0.263
59	Peptidyl-prolyl-cis-trans isomerase	P17742	4.668	-	-	4.668
**Cell structure proteins**						
65	Destrin	Q9R0P5	6.379	-	-	6.379
35	put. beta-actin	P60710	4.535	-	3.131	3.833*
22	Vimentin	P20152	0.007	0.020	0.759	0.262*
73	Desmin	P31001	0.062	0.028	0.092	0.061
28	Tubulin beta 5	P99024	2.086	-	1.674	1.880
67	Lamin B1	Q61791	0.388	0.556	0.545	0.496**
**Energy metabolism**						
26	Phosphoglycerate kinase 1	P09411	3.535	7.459	1.422	4.139***
37	Ubiquinol-cytochrom-c reductase core protein 1	Q9CZ13	0.634	0.335	0.926	0.632
47	Enolase 3, beta muscle	P13929	8.671	6.014	5.691	6.792*
54	Lactat dehydrogenase A	P06151	12.153	6.044	8.854	9.017**
48	Pyruvate kinase M	P52480				
55	Phosphoglyceratmutase 1	Q9DBJ1	1.823	1.955	1.581	1.786
56	Malat dehydrogenase, cytoplasm	P14152	7.549	7.815	3.969	6.444**
57	Triosephosphate isomerase (TIM)	P17751	9.144	6.492	9.204	8.280
64	Glycerinaldehyde-3-phosphate dehydrogenase	P16858	7.419	6.152	12.700	8.757***
77	Fructose-bisphosphate-aldolase 1	P05064	7.802	5.373	2.973	5.383**
3	Succinate dehydrogenase Fp subunit	Q8K2B3	0.471	0.376	0.548	0.465*
7	Alpha-glycosidase 2 alpha neutral subunit	Q8BHN3	0.487	0.439	0.486	0.471***
8	Aconitase 2, mitochondrial	Q99KI0	0.585	0.375	0.187	0.382
43	Dihydrolipoamide S-succinyltransferase	Q9D2G2	0.383	0.604	0.748	0.578
51	3-oxoacid CoA transferase 1	Q9D0K2	0.575	0.307	0.334	0.405
**Inflammation**						
9	Complement component 1, q subcomp. binding protein	Q8R5L1	1.565	1.679	1.020	1.421

a *^)^ Spot ID from* *Fig. 2A* *.*

b *^)^ Swiss-Prot accession.*

c ^)^ Fold change (exposed versus control) following induction of ischemia (n = 3, *p<0.05, **p<0.01 and ***p<0.001).

### Validation of the 2-DE results

**Figure 3:**
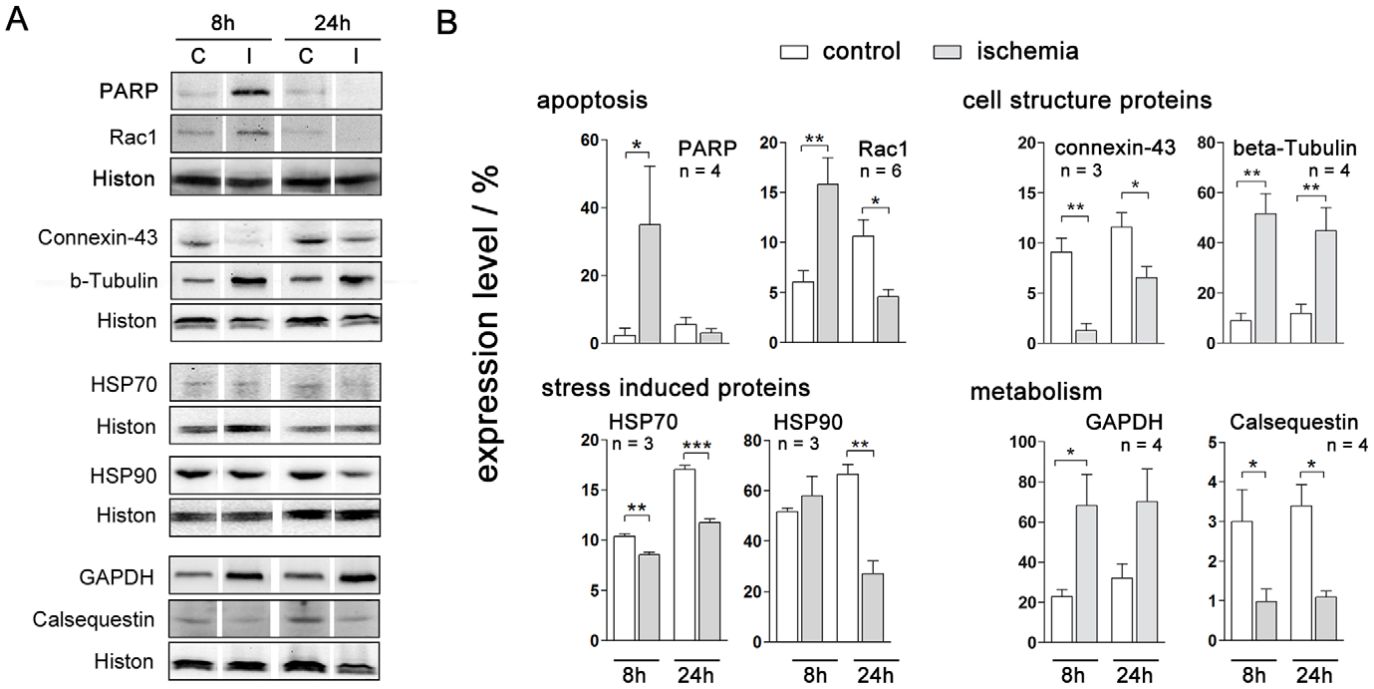
Cytosolic HL-1 cardiomyocyte proteins showing changes in expression after cellular exposure to ischemic conditions. (A) For four identified pathways verification was done by western blot analysis of two proteins per pathway. Shown is the expression in HL-1 cardiomyocytes after induction of ischemia (8 h) and after reperfusion (24 h) from control (C) and treated cells (I). (B) All protein expression levels were calculated relative to the housekeeping protein histone H3 and three to six experiments were statistical analysed.

From each of the four biological pathways, apoptosis, molecular chaperons, cell structure proteins and cell metabolism, we have initially chosen two representative proteins to validate 2-DE results by an independent method. Using SDS-PAGE and immunoblot for the detection of changes in protein expression after ischemia induction in HL-1 cardiomyocytes, we were able to confirm the results from 2-DE (Fig. 3). By analysing the caspase-3 substrate PARP, which is cleaved in a consequence of apoptotic cascade activation, we could distinguish an increase of cleaved-PARP in ischemic HL-1 cardiomyocytes eight hours after ischemia induction (5-fold) indicating that apoptosis plays a crucial role in ischemia, although revitalization leads to a decrease of cleaved-PARP in ischemic HL-1 cardiomyocytes. Also HSP70 and HSP90 expression correlates with the data observed from 2-DE. Whereas ischemic cells showed a slightly but not significant increase of HSP90 expression from 51.6±1.4% (control) compared to 58.0±7.6% (ischemia) after eight hours HSP70 decreased significantly from 10.4±0.2% (control) compared to 8.5±0.2% (ischemia). However, both heat shock proteins showed a significant decrease after reperfusion. The specific detection of decreasing connexin-43 pointed to a degradation of the cell-cell contacts and the cytoskeleton (7-fold down-regulated). Other cytoskeleton proteins, like beta-actin and desmin, were shown to be also down-regulated in the 2-DE. Finally, we were also able to demonstrate the activation of catabolic energy processes, like glycolysis enzymes, as a result of ischemia injury. Complete values are shown in Table S3.

### Effects of induced ischemia on cellular structure in the HL-1 cell monolayer

For further validation of 2-DE and western blot data, the expression and subcellular organization of the molecular maker proteins alpha-actinin, desmin and cadherin were investigated by immunocytochemical staining (Fig. 1). In control cells filaments and structural proteins were expressed constantly during the whole experiment, whereas ischemic HL-1 shows destruction in that, resulting in a cessation of contractility. The actin-crosslinking protein alpha-actinin is diminished in cardiomyocytes 8 h after ischemia induction. This effect is corrected after revitalization, where the expression of alpha-actinin occurs increased. Same effect could be observed in the immunocytochemical analysis of the intermediate filament desmin. Following 8 h incubation of HL-1 cardiomyocytes in ischemic buffer, desmin expression is decreased, whereas desmin level increases after revitalization period in Claycomb-medium. This cytoskeleton degradation indicates the reduction of contractility behaviours during ischemia, whereas rebuilding of cytoskeleton organisation after revitalization period recovering cardiomyocytes contractility. Beside this, observed shrinking of nuclei indicated the cell degradation during apoptosis.

Furthermore we could demonstrate a loss of cell-cell interactions in HL-1 cardiomyocytes in consequence of ischemia and apoptosis by immunocytochemical staining of the cell-connecting transmembrane protein cadherin. Cadherin expression in the cell-cell-interspaces of control samples specifies a well-connected cell layer. In contrast to that incubation in ischemic buffer led to a down regulation of cadherin, and disturbed contraction forwarding may be due to the resulting syncytium failure. However, cadherin expression and the formation of a large-area contractile monolayer recovered after revitalization.

### Effects of induced ischemia on proliferation and apoptosis in HL-1 cells

**Figure 4:**
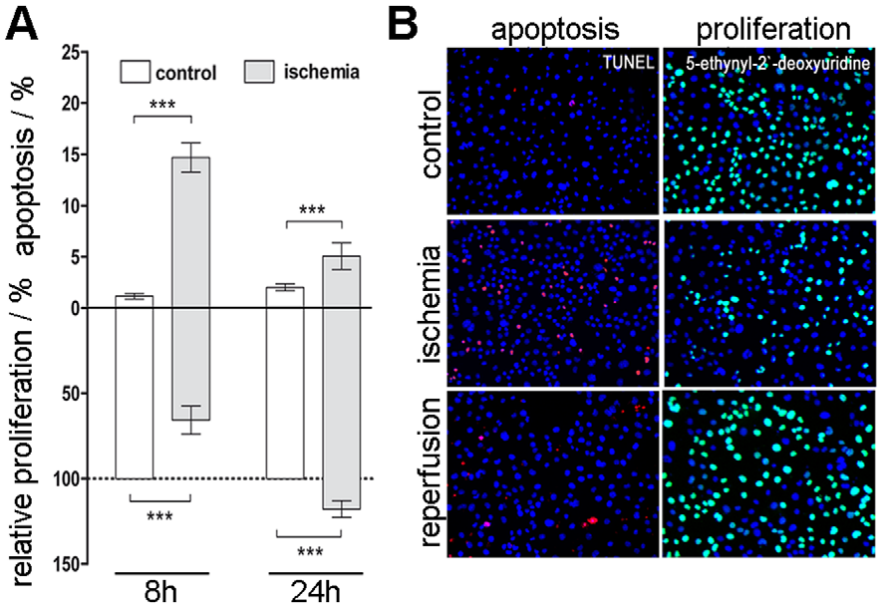
Apoptosis and proliferation analysis of ischemic HL-1 cardiomyocytes. Induced ischemia resulted in an increased apoptosis and decrease of proliferation. (A) For quantification apoptotic cells were stained using the TUNEL-assay, proliferating cells were labelled with EdU using the Click-iT™-assay and analysed via flow cytometer. For comparison of different time points, proliferation values were normalized to controls of the corresponding time point (n = 3). (B) Immunocytochemical staining of TUNEL positive apoptotic HL-1 cardiomyocytes (red) and EdU positive proliferating cells (green) in control cells, ischemic cells and reperfused cells. Total cell number obtained by a DAPI stain (blue). Scale bar = 100 µm.

Due to the fact, that ischemia and reperfusion time is strongly associated with apoptosis and changes in proliferation behaviours, treated HL-1 cells were analysed using TUNEL-apoptosis assay and Click-iT™ proliferation assay. The mean values and standard error of mean of three independent experiments were calculated for both applications and statistically analysed for significance (Fig. 4).

Flow cytometry analysis showed a significant increase of apoptotic cells after induction of ischemia; confirm the data from 2-DE and western blot analysis. While non-treated cells showed a considerably low percentage of apoptotic cells within the whole experiment (1.16±0.31% and 2.03±0.44%), incubation of HL-1 cells in nutrient deficiency medium led to an increase of apoptosis rate to 14.68±1.5% after 8 h. Even 16 h after revitalization in standard Claycomb-medium, the number of apoptotic cells were still significantly higher (5.07±0.62%) compared to control samples.

Proliferation analysis was done using the incorporation of 5′-ethynyl-2′-desoxyuridine (EdU) in genetic material during active DNA-synthesis. Due to different incubation times with EdU caused by the experimental protocol, all results have to be normalized to respective control values. However, incubation of HL-1 cells under ischemic condition led to a significantly decrease of relative proliferation to 65.61±14.26%, whereas revitalization of ischemic HL-1 cells in standard Claycomb-medium results in a mercurial increase of relative proliferation rate up to 117.7±8.5% indicating a nearly complete revitalization of ischemic cells.

### Differential protein expression in human cardiac tissue following ischemic cardiomyopathy

First experiments to analyse human proteome from patients with ischemic cardiomyopathies by DIGE 2-DE showed a high concentration of human serum albumin, which interfered with the adequate focusing and detection of lower abundance proteins (Fig. S3). Potential protein biomarkers are supposed to occur at concentrations up to 10 orders of magnitude less than the most abundant proteins. For biomarker discovery and subsequent biomarker evaluation it is essential to reduce the dynamic range [22], [23] of the most comprehensive proteome. Immunoaffinity-based depletion of high-abundant proteins has been described as the most specific method for removal of highly abundant proteins [24] and may increase the detection of the lower abundance proteins which otherwise would not be visible.

With the objection to reduce the complexity of biopsy proteome for subsequent proteomic 2-DE DIGE analysis we depleted high abundant albumin and IgG using monoclonal antibody strategy according to manufacturer's instruction (Qiagen). Following removal, samples were subjected to SDS-PAGE as a preliminary screen for efficacy and specificity of removal method, exposing almost complete depletion. Removal columns were essentially devoid of albumin and IgG and the albumin fractions contained predominantly the albumin line (Fig. S3A). To assess depletion rates Coomassie-stained gels were analysed using densitometry. Depletion averages at least 79.27±10.19%.

**Figure 5:**
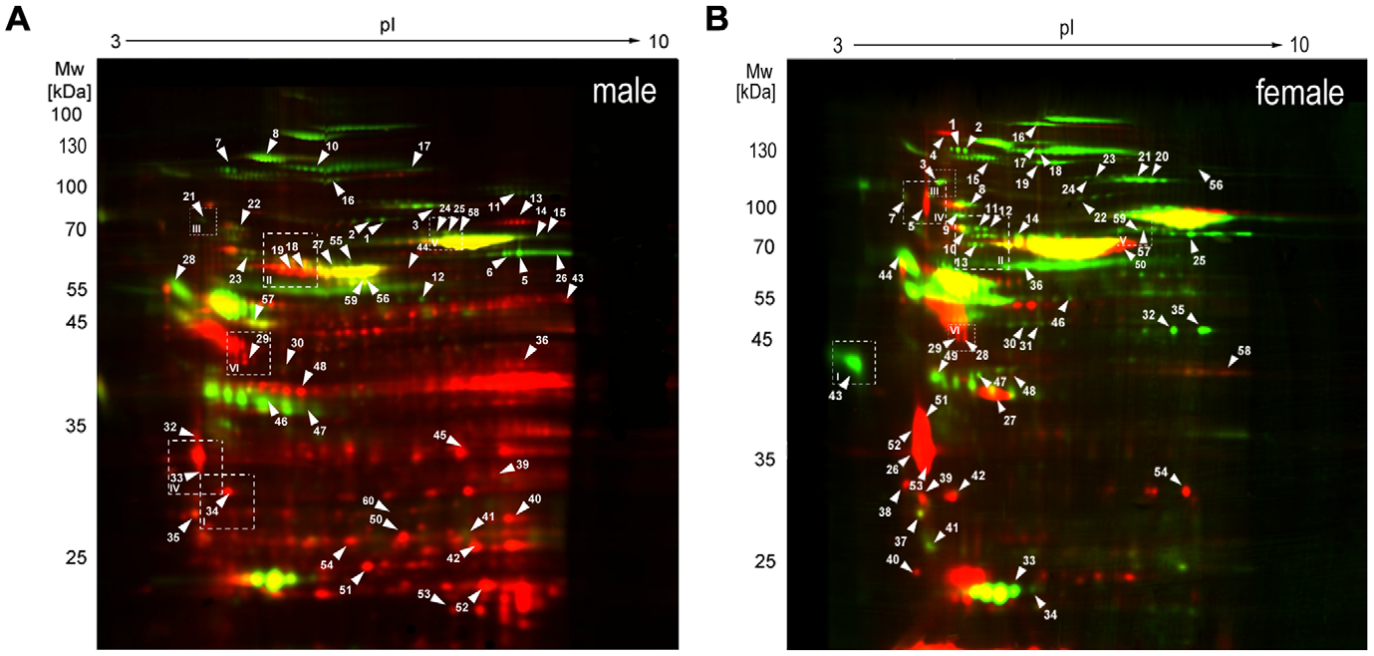
Proteome map of human biopsy samples from patients with ischemic cardiomyopathy. (Identification data are shown in Table S4 and Table S5). In total 360 µg protein of male (A) and female (B) biopsy samples were separated by DIGE-2-DE (18 cm IPG stripes 3–10 non-linear, 10% acrylamide/bisacrylamide).

The depletion of the high-abundant proteins albumin and IgG from human biopsy samples allowed the loading of 450 µg protein of the enriched fraction and thereby the detection of spots previously masked by high-abundance proteins in terms of mass and spot size. Especially albumin covered a large area on 2-DE gels of crude samples that could be used for resolving other spots after depletion. With the sample complexity reduced, and the detection range increased (Fig. S3B), we turned our efforts for protein identification in male and female human samples. Typically 2-DE spot patterns of male and female biopsies are shown in Fig. 5. For quantitative analysis digitalized fusion gel was composed of a total of three gels in with the Cy 5-labeled ischemia samples were compared to the Cy 3-labeled controls. In total 739 protein spots could be detected in the male fusion gel, and 962 spots were detected in the female fusion gel. We quantified the expression using Delta 2D software, version 3.6. In male 2-DE gels 171 protein spots (that equates 22.8% of all detected spots), in female 2-DE gels 341 spots (35.8%) displayed a difference in expression stronger than 2-fold with *p*<0.05, appeared in all of the three gel-replicates. The reproducibility of the 2-DE gels was confirmed by hierarchical cluster analysis. The comparison between the diseased and control samples showed a clear clustering of the gels according to the groups of samples (heatmaps are shown in Fig. S2). We investigated 60 of these spots per gender by tryptic digestion and MS analysis of the peptides. Finally, we could identify 53 male and 56 female significantly regulated spots using ESI-MS. Following ischemia injury 14 protein spots were up-regulated and 33 spots were down-regulated in male samples, 16 protein spots were up-regulated and 40 spots were down-regulated in female samples. Proteins could be classified in different cellular processes such as oxidative stress regulation, cell death signaling, inflammation, cytoskeleton organisation, protein quality control and metabolism (Table 2; all identified proteins were listed in Table S4 and Table S5).

**Table 2:** Annotation of identified proteins from differentially abundant spots following ischemic cardiomyopathy in biopsy samples to functional groups.

ID^a^	Protein involved in process	Gender	Accession^b^	Fold change^c^	
male/female				male	female
**Cell death signaling/apoptosis**					
34/43	Annexin A5	male/female	gi|809185	67.74**	0.12*
1	Serpin peptidase inhibitor, clade A	female	gi|15990507	-	0.06*
35	Tyr3/trp5 -monooxygenase activation protein, epsilon	male	gi|5803225	2.59	-
25	Cullin 1	female	giI21358757	-	0.12**
33	Rho GTPase activating protein 21	female	giI57162515	-	0.11**
40	EDAR-associated death domain, isoform CRA_b	female	giI119590454	-	5.86
56	Inhibitor of apoptosis protein1	female	giI110590599	-	0.15**
58	Apoptosis-inducing factor (AIF)	female	gi|14318424	-	0.21*
37	Chain A, 14-3-3 Protein Epsilon	female	gi|67464424	-	0.29
**Oxidative stress**					
44	Mit. aldehyde dehydrogenase 2 precursor variant	male	gi|62898307	122.34	-
40	Carbonic anhydrase I	male	gi|4502517	2.56	-
**Protein quality control**					
19/13,14	Heat shock 70 kDa protein 8 isoform 1	male/female	gi|5729877	6.58*	0.49*
21/3	Heat shock protein 90 kDa beta, member 1	male/female	gi|4507677	0.75	0.15
50	Heat shock protein beta-1 ( = HSP27)	male	gi|4504517	1.76	-
8	Heat shock protein HSP 90-alpha 2	female	gi|61656603	-	0.20
10	Heat shock 70 kDa protein 5	female	gi|16507237	-	0.75
46	Mitochondrial heat shock 60 kD protein 1 variant 1	female	gi|189502784	-	0.25
34	Peptidylprolyl isomerase E (Cyclophilin E)	female	giI62955333	-	0.10*
28	Calreticulin precursor	male	gi|4757900	0.20*	-
**Cell structure proteins**					
49	Beta actin variant	female	gi|194385944	-	0.25**
52	Tropomyosin beta, isoform 2	female	gi|47519616	-	76.21
33/5,26,54	Tropomyosin alpha, isoform 1	male/female	gi|49660014	596.89	34.92
53	Tropomyosin alpha, isoform 2	female	gi|63252902	-	292.74
27	Troponin T	female	gi|408217	-	43.09
38	Tropomyosin 4, isoform 2	female	gi|4507651	-	0.91
39	Tropomyosin 3, isoform 2	female	gi|24119203	-	4.40**
11	Intermediate filament protein	female	gi|28317	-	0.36*
**Inflammation**					
24,25,58/59	Transferrin	male/female	gi|4557871	0.31	0.69
5/22	Complement factor B	male/female	gi|291922	0.04*	1.79
8/19	Ceruloplasmin (ferroxidase)	male/female	giI11999289	0.06*	0.83
16/16	Complement factor H	male/female	gi|62739186	0.10	0.53
1	Alpha-1-antichymotrypsin	male	gi|177809	0.18*	-
9	Coagulation factor II preproprotein	female	gi|4503635	-	0.25*
23	C9 complement protein	male	gi|179726	0.32	-
**Energy metabolism**					
3	Gelsolin isoform a precursor	male	gi|4504165	0.10	-
14,15	Aconitase 2	male	gi|4501867	0.07*	-
29,30/28,29	Mit. ATP synthase, H+ transporting F1 complex beta	male/female	gi|89574029	1001.29	29.91*
42	Triosephosphate isomerase	male	gi|999892	10.56	-
44	Mit. aldehyde dehydrogenase 2 precursor variant	male	gi|62898307	122.34	-
45	Cytosolic malate dehydrogenase	male	gi|5174539	0.80	-
35	2-phosphopyruvate-hydratase alpha-enolase	female	gi|693933	-	0.04*
47	Creatine kinase B	male	gi|180555	0.09*	-

a *^)^ Spot ID from* *Fig. 2A* *.*

b *^)^ NCBI accession.*

c ^)^ Fold change (exposed versus control) following induction of ischemia (n = 3, *p<0.05 and **p<0.01).

Findings are in good agreement with the identified proteome data collected from *in vitro* HL-1 disease model. Proteins could be clustered into the same groups (Table 1 and Table 2), demonstration the suitability of our *in vitro* model system for simulating ischemic effects.

**Figure 6:**
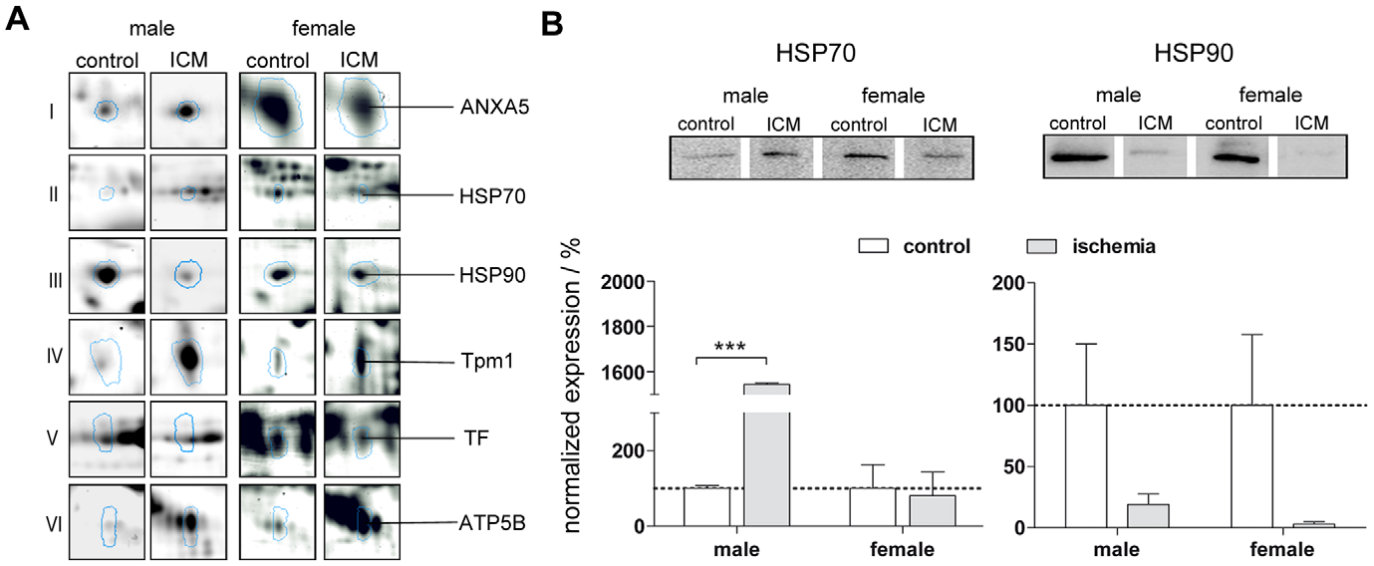
Gel image regions of differentially expressed proteins following ischemic cardiomyopathy in male and female biopsies. (A) Abbreviations are gene names. Regions are emphasized in the DIGE-2D-gel images in Figure 5. (B) Relative Expression of HSP70 (left) and HSP90 (right) in male and female biopsies by western blot analysis, normalized to control samples.

For validation of the biopsy proteome data, we analysed the heat shock proteins HSP70 and HSP90 by western blot analysis, which were significant differentially expressed in 2D-gels. Whereas HSP90 variants showed a decrease in diseased male and female samples (ratio of 0.75 and 0.15/0.2) the HSP70 protein 8 isoform 1 was up-regulated (ratio 6.58) in male, but down-regulated (ratio 0.49) in female samples. Same trend was observed in western blot analysis (Fig. 6B). Both gender showed a decrease of HSP90 level after ischemia injury with a ratio of 0.2 in male and 0.03 in female samples, but a different attitude in HSP70 expression. Whereas in male biopsy samples the HSP70 level increased up to the 15-fold of magnitude, HSP70 expression in female samples decreased to a ratio of 0.8. These data are in good agreement with the proteome data of heat shock protein 70 and 90.

## Discussion

Heart disease is the leading cause of mortality and morbidity in the world [25] and presently the most costly health care diagnosis. Due to the lack of effective tools, most early stage of heart disease is not detected clinically until irreversible tissue damage accrues, therefore the discovery of biomarkers allowing for early diagnosis and therapeutic monitoring of at risk patients is urgently needed. Proteomics is a powerful tool for biomarker discovery as well as target identification for novel drug design [11], because changes in global protein expression levels of cardiomyocytes in response to ischemic conditions may provide unique insight and understanding of the respective underlying mechanisms. Although the most suited medium for that would be blood samples it seems advisable to understand in more detail the changes in the affected tissue prior to tedious serum screening.

Proteomic studies of human heart tissue are complicated by factors such as disease state, tissue heterogeneity, genetic variability, medical history and therapeutic investigations [11].

To overcome these problems many animal models of ischemic disease have been established. With this work our aim was to develop a mouse myocardial infarction in *vitro* model to study cellular and molecular changes after induction of ischemia and ischemia/reperfusion, without any artificial influences of animal models like genetic diversity or retrovirus infection [26]. Therefore we applied the contractile cardiomyocyte cell line HL-1, which is the only cell line that contracts and maintains phenotypic and electrophysiological characteristics [17]. Due to the extensive changes caused by the induction of ischemic conditions also the abundances of proteins was altered rather dramatically. In general, more repressed than induced protein spots were identified in ischemic HL-1 cells, in which mainly degradation processes were involved, like decomposition of cytoskeletal proteins or cell-cell contact proteins. This reorganization was also detected by immunocytochemical staining of alpha-actinin, desmin and the cell-adhesion molecule cadherin (Fig. 1). 8 h after induction of ischemia degradation of cytoskeleton and a loss of cell-cell contact by degradation of the cell monolayer could be detected, what affects a cessation of contractile behaviours in HL-1 cells. The observed loss of contraction power and frequency was monitored via microscope and field potential measurement (Fig. S1) and was in concordance with former *in vivo* and *in vitro* studies [27]. Additionally, ischemia causes a lack of energy. For example, in the ischemic heart, a reduction in mitochondrial oxidative capacity serves to reduce oxygen consumption in the context of limited O_2_ availability. Reduced capacity for energy transduction leads to deregulation of cellular processes critical for cardiac pump function, including Ca^2+^ handling and contractile function [15], [28]. In the ischemic state many proteins involved in glycolysis were increased, whereas proteins involved in energy consuming pathways were decreased, pointing to a counteractive mechanism. This finding is in agreement with previous studies [29]. Moreover, proteins which reveal an induction of apoptosis in cellular response due to ischemia conditions were detected. In this context, early apoptosis promoting mechanisms were found to be activated. Vinculin, granzyme k and annexin A5 were up-regulated, whereas nucleophosmin 1, a protein that prevents apoptosis [30], [31] and inhibit the caspase-activated DNase [32] was down-regulated in all three replicates. Immunocytochemical staining of the cytoskeleton components (Fig. 1) showed also shrunken nuclei, indicating apoptotic cells. Western blot analysis as well as flow cytometric analysis revealed an increase of apoptosis by ischemia induction, too. These findings are in line with earlier studies that showed an increased apoptosis rate in cardiomyocytes in consequence of myocardial infarction [33], [34]. Furthermore, the observed decrease of proliferation is prominent in cardiac infarct and its implications. However, by induction of reperfusion in HL-1 cardiomyocytes using Claycomb-culture medium cells increased proliferation behaviours, displaying regeneration in cell vitality. After reperfusion phase proteome alterations approximate to control samples. There were significantly less changes in reperfused than in ischemic 2DE-gels. Mostly, proteins involved in cell death signals or oxidative stress regulation were detected, what is in accordance with former studies of cardiac remodelling after acute myocardial infarction [15], [35].

To validate the results of our HL-1 disease model human biopsy samples, collected from patients with ischemic cardiomyopathy were analysed via 2D-GE and compared to the ischemic *in vitro* samples.

One major challenge in functional proteomics is the separation of complex samples prior to comparative analysis e.g. for disease marker identification. This applies especially for blood samples but was also found to happen in case of the biopsy samples from heart tissue. For this reason we applied the depletion of the high abundant serum proteins albumin and IgG using the Depletion kit from Qiagen and achieved a diminution of 79.27±10.19%, increasing the detection of the lower abundance cytosolic proteins which otherwise would not have been visible. Thus we were able to identify changes in the proteome from male and female patients with ischemic cardiomyopathy. Identified proteins agreed with published proteome data taken from human DCM (dilated cardiomyopathy) and ischemic animal models [11]. Standard two-dimensional gels can routinely separate 1,000 to 2,000 proteins from a total cardiac extract [36]. However, it is clear that this gives incomplete proteomic coverage for the human heart, which may express more than 10,000 proteins [11]. Nonetheless, we detected cytoplasmatic proteins, which are involved in relevant pathological processes. Troponin T has been demonstrates as most reliable to detect and estimate the extent of cardiac damage [37]. Apolipoprotein E, tropomyosins, alpha-2 macroglobulin, complement 3, creatin kinase B and ceruloplasmin have been also described as biomarkers for chronic heart failure [38]–[42]. Comparing the identified proteins from the biopsy material with the in vitro model, we are aware that a 24 hour *in vitro* model never could be completely comparable to a pathological *in vivo* situation that arises over years or even decades. So the aim of our study was first to identify the main altered pathways, not single proteins in our 24 hour in vitro ischemic cardiomyopathy model and second to validate it with ischemic cardiomyopathy biopsies from patients. Therefore, we could demonstrate a correlation of identified pathway alterations. So we are absolutely convinced that our developed and analyzed *in vitro* model could be useful to overcome the dilemma of the research community and especially of the pharmaceutical industry, where easy to handle standardized culture models with fast developing pathology are needed (e.g. for the active pharmaceutical ingredients development).

Furthermore, we were able to demonstrate a differential protein expression pattern from male and female proteome of patients with ischemic cardiomyopathy. Thus a gender specific expression of HSP70 was observed in human biopsy samples. Whereas in male proteome HSP70 was significant up-regulated, HSP70 was down-regulated in female samples (Fig. 6A). This finding is of great importance showing that for individual therapy target a gender specific treatment have to be taken into account. Beside this gender specific single protein expression differences, in general our investigation of male and female biopsies revealed common alterations in the biological pathways, such as oxidative stress mechanisms, apoptosis and cell death, cytoskeletal reorganization, energy metabolism and Ca^2+^-homeostasis as identified by database search and peer reviewed journal screening.

In addition to the identified proteins with altered expression post-translational modifications (PTMs) are important for the regulation of functional activities of proteins. PTMs such as phosphorylation or acetylation could be identified in the imaged 2-DE gels as “train of spots”.

Unfortunately, the search for phosphorylation and acetylation on the basis of the obtained spectra we could not unambiguously identify any of those PTMs. Since PTMs were not the main intension of this study, we didn't investigated in PTMs by further experiments. But having now a validated *in vitro* ischemia model we can use it now for further studies also with regard to PTMs.

### Conclusion

Demonstrating the suitability of our mouse myocardial infarction model for studying cellular and molecular changes after induction of ischemia and ischemia-reperfusion *in vitro* we were able to illustrate similar alterations in the proteome as seen in pathological biopsies taken from patients with ischemic cardiomyopathy concerning proteins of cell death pathways and oxidative stress response. Our *in vitro* model opens up the path to investigate cellular mechanisms that are involved in ischemia and cardiac dysfunctions recapitulating major pathway alterations close to the *in vivo* situation and thereby offers the chance to identify new diagnostic and therapeutic markers.

## Materials and Methods

### Ethics Statement

The collection of human cardiac tissue samples used in this study was approved by the Ethics Committee of the Medical University of Vienna, and individuals who signed the written informed consent to be enrolled were included between January 2004 and July 2008.

### Cell culture and induction of ischemia *in vitro*

The HL-1 cardiomyocytes (murine atrial tumor cell line) were maintained in monolayer culture with Claycomb-medium (Sigma, Germany), supplemented with 10% fetal calf serum (SAFC-Biosciences, USA), 1% penicillin/streptomycin, 1% glutamax (both Invitrogen, Germany) and 1% norepinephrine (Sigma-Aldrich, Germany) in an incubator containing 5% CO_2_ at a relative humidity of 95% and 37°C. 8×10^5^ cells/cm^2^ were seeded in supplemented Claycomb-medium on fibronectin/gelatine-precoated cell culture flasks. Coating was performed using a 1 mg/ml fibronectin-solution in 0.02% gelatine (Sigma-Aldrich, Germany) and incubate at 37°C overnight. The Claycomb-medium, which is specifically designed for the growth of HL-1 cells, was replaced approximately every 48 h. Heaving reached confluence and contractile activity, cells were maintained as subcultures using 0.05% trypsin/EDTA (Invitrogen, Germany) and subsequently incubated under standard culture conditions.

Induction of ischemia was carried out on vital cardiomyocytes at culture day four. The subconfluent, contractile HL-1 cardiomyocytes were placed in nutrient-deficiency medium (136 mM NaCl, 5.4 mM KCl, 1 mM CaCl_2_, 0.53 mM MgCl_2_ and 5.5 mM HEPES in aqua dest., pH 6.7), containing 2.5 mM hydrogen peroxide solution (Merck, Germany) in order to enhance the oxidative stress in HL-1 cells. In control cultures the medium exchange was carried out with standard supplemented Claycomb-medium. 8 h after ischemia induction samples were harvested and revitalization was induced in parallel by replacing nutrient-deficiency medium with fresh Claycomb-medium and incubating cells for another 16 hours.

### Immunocytochemistry

For indirect immunofluorescence, HL-1 cells were grown on fibronectin/gelatine-coated glass coverslips, harvested at appropriate culture stages, and fixed with 4% formaldehyde in phosphate buffered saline for 15 min at room temperature. After repeated washing and dehydration, glass coverslips were stored at −20°C until use.

For immuncytochemical staining frozen glass coverslips were dried for 15 min at 37°C on a hotplate. Then cells were permeabilised by incubation with 3% BSA/0.1% Triton X-100 in PBS for 45 min at room temperature. The primary antibodies used for immunofluorescent staining were: monoclonal mouse-anti-α-actinin (Sigma- Aldrich, Germany) in a dilution of 1∶500, polyclonal rabbit-anti-desmin (1∶100; Dianova, Germany) and monoclonal mouse-anti-cadherin (1∶100; Abcam, UK). The secondary antibody was either Cy2-conjugated goat-anti-mouse IgG- or goat-anti-rabbit IgG. Nuclei were stained with Sytox®-orange (1 µM, Invitrogen, Germany).

The cultures were examined using a Nikon TE2000 fluorescence microscope equipped with a confocal laser scanning unit and documented with the EZ-C1 software.

### Proliferation and apoptosis assay

Cell proliferation analysis was done by flow cytometry via *Click-iT™-EdU* assay as described in detail in the manufacturer's instruction (Invitrogen). For labeling cells with EdU (5-ethynyl-2′-desoxyuridine, Invitrogen/Karlsruhe) the nucleoside analogue was added to the medium in a final concentration of 10 µM 8 h and accordingly 16 h before harvesting treated and non-treated HL-1 cells. Apoptosis analysis was performed by terminal desoxynucleotidyl transferase-mediated dUTP nick end-labeling using the *Click-iT™ TUNEL Alexa Fluor-488* assay (Invitrogen). Ischemic/reperfused and non-ischemic cells were collected using 0.25% trypsin/EDTA and fixed in 4% formaldehyde solution for 30 min at room temperature. Afterwards cells were kept in 0.1% triton/PBS until cell staining and flow cytometry with the FACS analyser FACS-Calibur (BD Biosciences, USA). Total number of cells was determined using 7-AAD nucleus staining (0.05 µg/µl; BD Biosciences, USA).

Additionally formaldehyde-fixed cells on glass coverslips were prepared for immuncytochemical staining as described in section 2.2. TUNEL assay was performed using the *In Situ Cell Death Detection Kit TMRred* (Roche Diagnostics, Mannheim/Germany), whereas Proliferation behaviour was analysed via *Click-iT™-EdU* assay (Invitrogen). Nuclei were stained with DAPI (0.1 mg/ml, Sigma-Aldrich) to detect the total number of cells. Cultures were examined and documented with a Nikon Eclipse 200 fluorescence microscope with the LUCIA 4.8 software.

### Human myocardial samples and protein isolation

Human biopsy materials were received as protein lysates from University hospital of Vienna. The myocardial biopsies were collected from explanted hearts of patients (3 males, 3 females, age 44–61 years) with ischemic cardiomyopathy (ICM) undergoing cardiac transplantation according to the American Heart Association criteria as referred to earlier [43]. The control group consisted of 6 heart donors (3 males, 3 females, age 31–47 years old) with no history of cardiac disease whose hearts could not be transplanted due to quality reasons. The inclusion and exclusion criteria of patients have been performed as described [44]. All patients underwent an optimized heart failure treatment prior to transplant, including angiotensin-converting enzyme inhibitors, angiotensin II receptor blockers, beta blockers, statins, sedacoron, digitalis, coumarin and prostaglandin. After obtaining informed consent, myocardial biopsies were dissected 20 mm distal to the diagonal branch and 20 mm from the left anterior descending coronary artery from the left ventricle of the explanted hearts as described elsewhere [45]. Samples were coded, snap frozen and stored in liquid nitrogen until further use.

For protein isolation the myocardial biopsies were homogenized in Flackelton Lysis buffer, containing PMSF (1 mM), Na_3_VO_4_ (100 mM) and EDTA-free protease inhibitor cocktail (4%, Roche) and the cytosolic fraction of protein extracts was stored at −80°C.

### Albumin and IgG depletion in human protein samples

Serum albumin and IgG were depleted from protein samples by applying the Albumin and IgG Depletion Kit *Qproteome™* (Qiagen, Germany) using immunoaffinity-based depletion spin columns. Control protein samples were mixed 1∶1, ischemic biopsy samples 9∶1 with 20 mM HEPES buffer (Merck; pH 7.2), and applied onto the pre-equilibrated columns. Columns were shaken vigorously to obtain a homogenous suspension and incubated for 5 min on an end-over-end shaker at room temperature. Flow through, containing the depleted protein sample, was collected by centrifugation at 500× g for 10 s. After washing columns with 2×100 µl aliquots of HEPES buffer and centrifugation at 500× g for 10 s, through-flow fraction and wash-fractions were combined and protein concentration was determined according to the method of Bradford. For visualization the depletion efficiency and to control unspecific binding, bounded albumin and IgG was removed from the resin using laemmli-buffer (62.5 mM tris-buffer, 2% SDS, 25% glycerol; pH 7.6) and all samples (pure sample, depleted sample and eluted albumin- and IgG-fraction) were separated by SDS-PAGE and stained with Coomassie brilliant blue G-250 (Merck, Darmstadt/Germany). Effectively depleted biopsy samples were then characterized by 2D DIGE (Fig. S2).

### Sample collection and preparation for proteome analysis

To harvest HL-1 cells for protein isolation, ischemic and non-ischemic cultures were detached 8 h and 24 h after induction of ischemia using a cell-scraper. After centrifugation (500× g, 5 min, 20°C) cells were washed twice in 0.5× PBS and stored at −80°C. For cell disruption cell pellet was resuspended in 100 µl homogenization-buffer (1 mM NaHCO_3_, 0.2 mM MgCl_2_, 0.2 mM CaCl_2_, 1 mM Spermidin, pH 8) including 1% proteinase inhibitor cocktail (Sigma-Aldrich, Germany) and sonicated on ice with an ultrasonic processor sonifier (Hielscher GmbH, Stuttgart/Germany; two cycles of sonification, 30 s each). Cell debris was removed by centrifugation (10 000× g, 10 min, 4°C) and the protein concentration of the supernatant was determined according to the method of Bradford. The isolated proteins were stored at −80°C. Biopsy samples were also sonicated as described and concentrations were sized by Bradford-assay.

150 µg of each sample were precipitated by adding five volumes of ice-cold acetone for 10 min at −20°C and centrifugation (13 000 rpm, 4°C, and 15 min). The resulting protein pellet was air dried, dissolved in 30 µl DIGE labelling-buffer (8 M urea, 30 mM Tris, 4% CHAPS, pH 9) and mixed for 10 min at 20°C.

### Protein cyanine dye labelling- DIGE

Cell lysates were labelled with N-hydroxy-succinimidyl ester-derivatives of the cyanine dyes Cy2, Cy3 and Cy5 (GE-Healthcare, Uppsala/Sweden) according to the recommendation of the supplier and as described earlier [46]. In brief, 1 nM stock solution of these dyes was diluted 1∶5 with dry DMF. If necessary, the pH of the protein solution was adjusted to pH 8.5 with DIGE-labelling buffer (pH 9), and then 100 µg of lysate was minimally labelled with 200 pmol of either Cy3 or Cy5. The labelling strategy was as follows: the control samples were always labelled with Cy3, the ischemic samples with Cy5 and for normalization the same amount of proteins from a pool of all samples was labelled with Cy2. Labelling reactions were performed on ice in the dark for 30 min and then quenched by addition of 0.5 µl of quenching solution (10 mM lysine; GE Healthcare, Uppsala/Sweden) to the labelling reaction followed by incubation for 10 min on ice. Differentially labelled samples were mixed prior to the application to 2D-gel electrophoresis.

### IPG-strip rehydration, IEF and 2D-gel electrophoresis

The mixed labelled samples were made up to 400 µl with DeStreak-rehydration solution (GE-Healthcare, Uppsala/Sweden), including 0.5% IPG buffer 3–10 NL and shaken for 10 min at 20°C. To remove unsolved proteins samples were centrifuged for 30 min, 13 000 rpm at 20°C. The protein solutions were applied to IPG-strips (18 cm, 3–10 NL, GE Healthcare) in the rehydration tray and overlaid with 3 ml oil. One IPG strip was used for each sample set, composed of Cy3 labelled control sample, Cy5 labelled ischemic/ischemic-reperfusion sample and Cy2 labelled pool of all samples. Immobilized non-linear pH gradient strips were rehydrated with Cy-labelled samples in the dark at room temperature overnight.

On the next day, each IPG strip was placed in the IEF-chamber (Ettan IPGphor 3, GE Healthcare). Isoelectric focussing started at 500 V and the voltage was gradually increased to 8000 V within 5 h and kept at 8000 V for at least 10 h at 20°C. The electrofocussing was finished after a total amount of 90–100 kVh. Following focussing and washing, strips were equilibrated on a shaker for 15 min with 2 ml equilibration buffer, containing 6 M urea, 30% glycerol, 4% SDS, 0.05 M Tris/HCl, bromophenol blue and 20 mg/ml DTE, followed by additional incubation for 15 min in the same buffer containing 25 mg/ml iodacetamide.

Second dimension electrophoresis was performed on a 10% SDS-PAGE gel. Therefore equilibrated IPG strips were transferred onto an 18×20 cm gel. Strips were covered with 1% agarose and run at 6 mA per gel at 12°C overnight until the bromophenol blue dye front just run on the base of the gel. After fluorescent scan, gels were stained with colloidal Coomassie brilliant blue G-250 (Merck, Darmstadt/Germany) and dried between polyethylene films.

### Quantitative gel analysis

Gel pictures were scanned using the Ettan DIGE imager II (GE Healthcare) and analysed in Decodon Delta-2D software vision 3.5 (Decodon, Greifswald/Germany) based on standard position computing and image fusion functions. After warping the gels using the all-to-one strategy, a fusion gel was created including all gels of the experiment. Spot detection was manually edited in the fusion gel and transferred then to all gel pictures. Normalization of spot volumes was done to the total protein amount on each gel (excluding the biggest spots representing ∼5% of total intensity) as described earlier [47]. Mean relative volumes of identical spots in triplicate gels were calculated and divided by the mean relative volume of the corresponding spots in the controls, yielding the expression ratio. Differentially expressed proteins were identified using the following parameters: expression ratio lowers than 0.5 or higher than 2 with a *p*-value of *p<0.05*, as obtained by the software's integrated Student's *t*-test.

### Analysis of peptides and identification of proteins

#### In gel digestion

Significantly up- or down-regulated protein spots were excised from dried colloidal Coomassie stained 2D-gels using a manual spot picker. Gel pieces were transferred to Eppendorf tubes and washed twice in a solution of 50% methanol, 5% acetate and 45% aqua dest.. The digestion was performed as described elsewhere [48]. In brief, gel pieces were incubated on a shaker in acetonitrile for 5 min and dried in a SpeedVac. Samples were reduced in 10 mM dithiotheritol in 10 mM ammonium bicarbonate for 30 min and then alkylated in 100 mM iodacetamide/10 mM ammonium bicarbonate for 30 min at room temperature. Then the gel pieces were washed twice in acetonitrile and 10 mM ammonium bicarbonate and vacuum dried. Digestion was done with sequencing grade-modified trypsin (Sigma-Aldrich) at 37°C overnight. After digestion, products were recovered by sequential extraction with 5 mM ammonium bicarbonate and extraction-buffer (50% acetonitrile, 44% aqua dest. and 6% of 85% formic acid). Peptide extracts were vacuum-dried and resuspended in 10 µl of 0.1% trifluoroacetic acid (TFA).

#### MALDI-TOF/TOF-MS and nano-LC-ESI-MS analysis

Digested HL-1 protein samples (0.5 µl) were spotted onto a MTP AnchorChip TM 600/384 MALDI plate (Bruker Daltonics, Bremen/Germany) and mixed with 1 µl of matrix (2.5 mg/ml α-cyano-4-hydroxy-cinnamic acid dissolved in acetonitrile/TFA; 50%/0.1%, v/v). The mixture was allowed to crystallize at room temperature. After washing the spots with 10 mM ammonium phosphate/0.1% TFA, recrystallization-solution (60% ethanol, 30% acetone, 0.1% TFA) was added and allowed to dry. MALDI-MS analysis was performed using the Ultraflex-III (Flex Control and Flex Analysis; Bruker Daltonics, Bremen/Germany). The instrument was calibrated using peptide calibration mix II as an external standard in MS and MS/MS procedure. The peptide calibration mix contained the following peptides: Angiotensin II, Angiotensin I, Substance P, Bombesin, ACTH clip 1–17, ACTH clip 18–39, Somatostatin 28 and covered thereby the mass range from 1000–3200 Da. Human biopsy samples were analysed with ESI-MS. Therefore, digested samples were desalted and purified using *ZipTip* strategy (Millipore; Billerica/USA) and dissolved in ESI-buffer containing 3% acetonitrile and 0.1% formic acid in aqua dest.. Peptides were separated by reversed-phase nano-LC (LC1100series, Agilent Technologies, Santa Clara, CA, USA; column: Zorbax 300SB-C18, 3.5 µm, 150×0.075 mm; eluent: 0.1% formic acid, 0–60% CAN [49]) and analysed by MS/MS TRAP XTC mass spectrometer, Agilent Technologies.

#### Database search

Database search was carried out using the MS/MS ion search (MASCOT 2.2.1, http://www.matrixscience.com) against either mouse for *in vitro* experiment samples or human for biopsy samples in the NCBInr database using the following parameters: trypsin digestion, up to one missed cleavage, fixed modifications: carbamidomethyl (C), and with the following variable modification: oxidation (M), mass tolerance 100 ppm. Proteins were specified as clearly identified if the Mowse score was higher than 55 (MALDI-MS) or 100 (ESI-MS) and at least two different peptides (*p*<0.05) were used for identification. Calculated molecular weight and p*I* were cross checked with the gel position of the excised spot. Identified proteins were categorized into discrete pathways by database research (pubmed) and peer reviewed journal screening.

### Western blot analysis

Equal amounts of protein from control and ischemic/ischemic-reperfused HL-1 cells, as well as control and diseased human biopsy samples were applied to 7.5–12% acrylamide gels and electrophoresed under reducing conditions at 100 V. After electrophoresis, samples were transferred onto PVDF membranes using a semi dry system (Bio-Rad). HSP70-specific polyclonal rabbit antibody and HSP90 specific monoclonal rabbit antibody (both 1∶1000; Cell Signaling Technology, Danvers/USA) for HL-1 cardiomyocytes and human biopsy proteins as well as rabbit polyclonal anti-PARP antibody (1∶1000; BD Pharmingen, USA), mouse monoclonal-rac1 antibody (1∶1000; Abcam, USA), rabbit polyclonal anti-Connexin-43 (1∶1000; Santa Cruz Biotechnology), rabbit polyclonal-anti calsequestrin (1∶1000; Abcam), rabbit polyclonal anti-beta-tubulin and rabbit polyclonal anti-GAPDH (both 1∶2000; Abcam) for the in *vitro* HL-1 culture were used for overnight incubation of PVDF-membranes. The antigens were detected using peroxidase-conjugated anti mouse- or anti rabbit antibody (0.08 µg/ml; Jackson ImmunoResearch, Suffolk/UK). Chemiluminescence signal was measured using Quantity One 4.3 measurement software (Bio-Rad). All measurements were normalized to histon signals using rabbit polyclonal anti histon h3 antibody (1∶5000; Abcam, Canada). Biopsy samples were normalized to the total protein amount.

### Electrophysiological measurement

For electrophysiological field potential measurements HL-1 cells were seeded on multielectrode-arrays (MEA) that consists of 60 titan-nitride microelectrodes with a diameter of 30 µm and an electrode spacing of 200 µm. When the HL-1 monolayer reached confluence and formed a synchronous beating syncytium, the MEAs were inserted into a MEA1060-BC field potential amplifier (Multichannel Systems, Germany) and incubated at 37°C for at least one hour prior to experiment beginning. To avoid evaporation of the medium by air-circulation arrays were covered with a cap. Field potential streams were recorded with 4 kHz sampling frequency. Signals were recorded for 8 h under ischemic conditions following revitalization with Claycomb-culture medium and recording for additionally 16 h. Detection of the electrode spikes was done offline by a self-developed software written in *MATLAB* version 7.01 (The Mathworks, USA).

### Statistical analysis

All statistical analyses were performed using GraphPad Prism 5. All values are expressed as means ± standard error of mean. Comparison between two groups was done by a two-tailed unpaired t-test. Comparison of two or more groups and parameters were done by 2D-ANOVA and Bonferroni post-hoc test. Proteins spots were statistical analysed using the Delta 2D version 3.6 integrated analysis tool incorporates algorithms from the TIGR Multiple Experiment Viewer (MeV, version 4.0, tm4.org/mev.html). Differences between two means with p<0.05 were considered significant, p<0.01 very significant and p<0.001 extremely significant.

## Supporting Information

Figure S1 **Experimental protocol and ischemia/reperfusion influence to contraction rate of HL-1 cardiomyocytes.** Cardiomyocytes were cultivated 4 days in *vitro* to a confluent and contractile phenotype, before induction of ischemia. After 8 h incubation medium exchange was done for revitalization of cardiomyocytes. Harvesting cells for proteomic and immunocytochemical applications was done before induction of ischemia (0 h), 8 h after induction of ischemia and 16 h after reperfusion (24 h). Contraction rate in ischemic and reperfused HL-1 cardiomyocytes was recorded by multielectrode-array based field potential measurement.
(TIF)

Figure S2 **Depletion efficiency of albumin and IgG removal in human biopsy samples.** (A) SDS-PAGE of biopsy samples before and after albumin and IgG removal using monoclonal antibody strategy. Lane 1: crude biopsy samples, lane 2: depleted samples, lane 3: albumin fractions. All fractions were separated on a 12% polyacrylamide gel and stained with Coomassie brilliant blue. Quantification was done by densitometry. (B) 2D-geleletrophoresis of crude biopsy samples (left) and depleted samples (right) stained with Coomassie brilliant blue (top images) and with Cy™-dyes using DIGE-strategy (bottom images). The albumin spot (white sphere) prior depletion disguises a lot of other spots, which could be detected sensitively after purification.
(TIF)

Figure S3 **Expression profile of analysed DIGE-2D-data from ischemic HL-1 cardiomyocytes and from biopsy samples from patients with ischemic cardiomyopathy via hierarchical cluster analysis.** By using an unsupervised approach comprising all spots the gels were clustered into the correct groups (n = 3).
(TIF)

Table S1 **Identified proteins from differentially abundant spots following ischemia in HL-1 cardiomyocytes.**
(DOC)

Table S2 **Identified proteins from spots of differentially abundant spots following reperfusion in HL-1 cardiomyocytes.**
(DOC)

Table S3 **Westernblot analysis of regulated proteins following ischemia/ischemia-reperfusion injury in HL-1 cardiomyocytes (% to histone H3).**
(DOC)

Table S4 **Identified proteins from differentially abundant spots following ischemic cardiomyopathy in male biopsy samples.**
(DOC)

Table S5 **Identified proteins from differentially abundant spots following ischemic cardiomyopathy in female biopsy samples.**
(DOC)
