# Supplementary figures and images for: DIGE Proteome Analysis Reveals Suitability of Ischemic Cardiac In Vitro Model for Studying Cellular Response to Acute Ischemia and Regeneration
Source: PLoS One. 2012 Feb 22;7(2):e31669. doi: 10.1371/journal.pone.0031669 (PMC3285183; doi:10.1371/journal.pone.0031669)

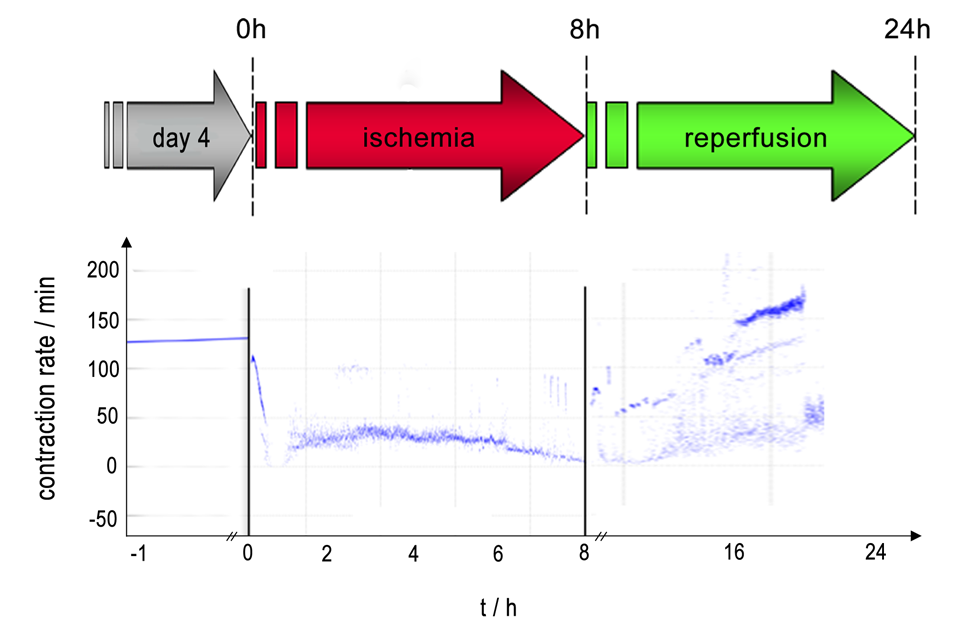

Supplement: Figure S1 — Experimental protocol and ischemia/reperfusion influence to contraction rate of HL-1 cardiomyocytes. Cardiomyocytes were cultivated 4 days in vitro to a confluent and contractile phenotype, before induction of ischemia. After 8 h incubation medium exchange was done for revitalization of cardiomyocytes. Harvesting cells for proteomic and immunocytochemical applications was done before induction of ischemia (0 h), 8 h after induction of ischemia and 16 h after reperfusion (24 h). Contraction rate in ischemic and reperfused HL-1 cardiomyocytes was recorded by multielectrode-array based field potential measurement. (TIF) [file pone.0031669.s001.tif]

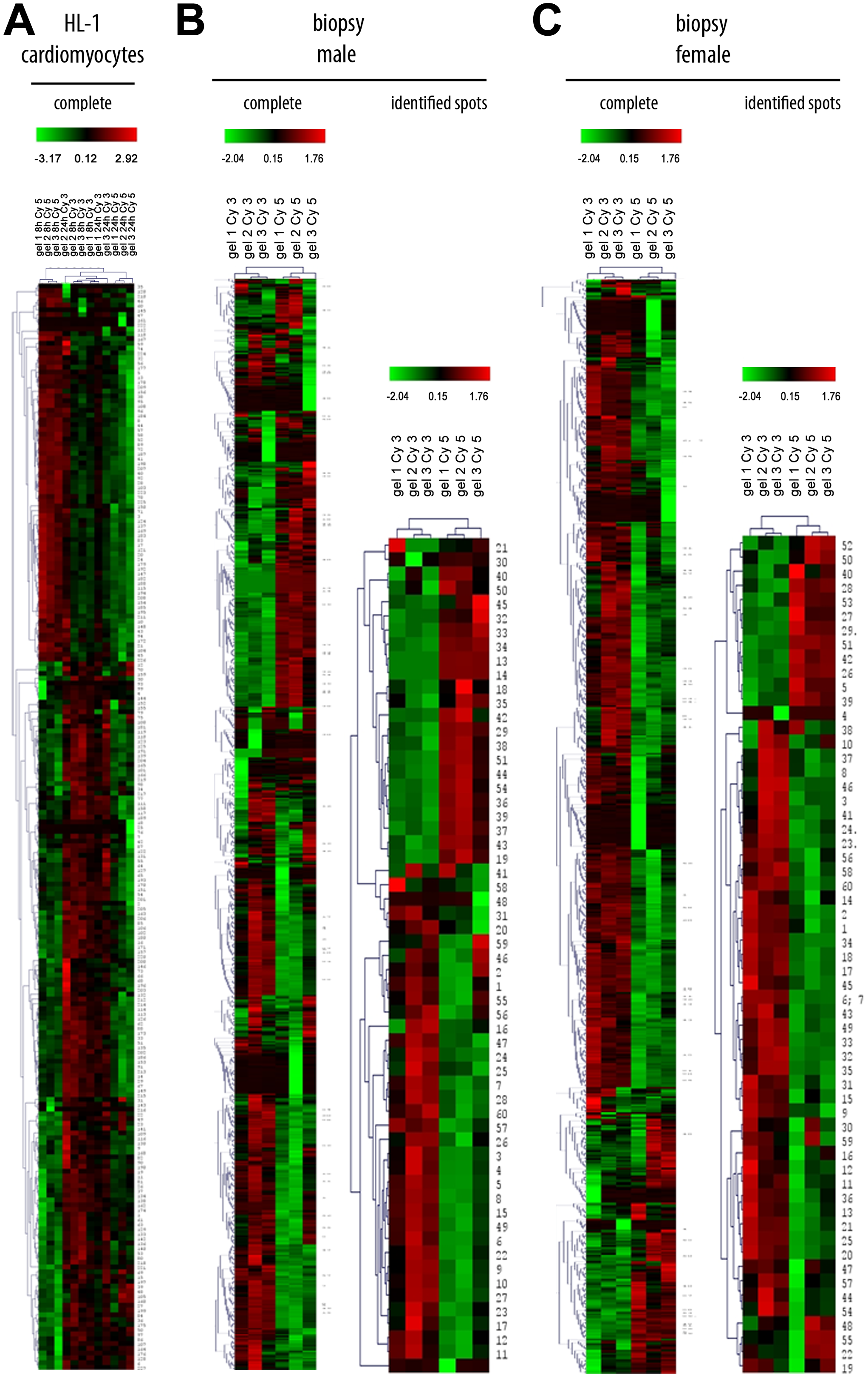

Supplement: Figure S2 — Depletion efficiency of albumin and IgG removal in human biopsy samples. (A) SDS-PAGE of biopsy samples before and after albumin and IgG removal using monoclonal antibody strategy. Lane 1: crude biopsy samples, lane 2: depleted samples, lane 3: albumin fractions. All fractions were separated on a 12% polyacrylamide gel and stained with Coomassie brilliant blue. Quantification was done by densitometry. (B) 2D-geleletrophoresis of crude biopsy samples (left) and depleted samples (right) stained with Coomassie brilliant blue (top images) and with Cy™-dyes using DIGE-strategy (bottom images). The albumin spot (white sphere) prior depletion disguises a lot of other spots, which could be detected sensitively after purification. (TIF) [file pone.0031669.s002.tif]

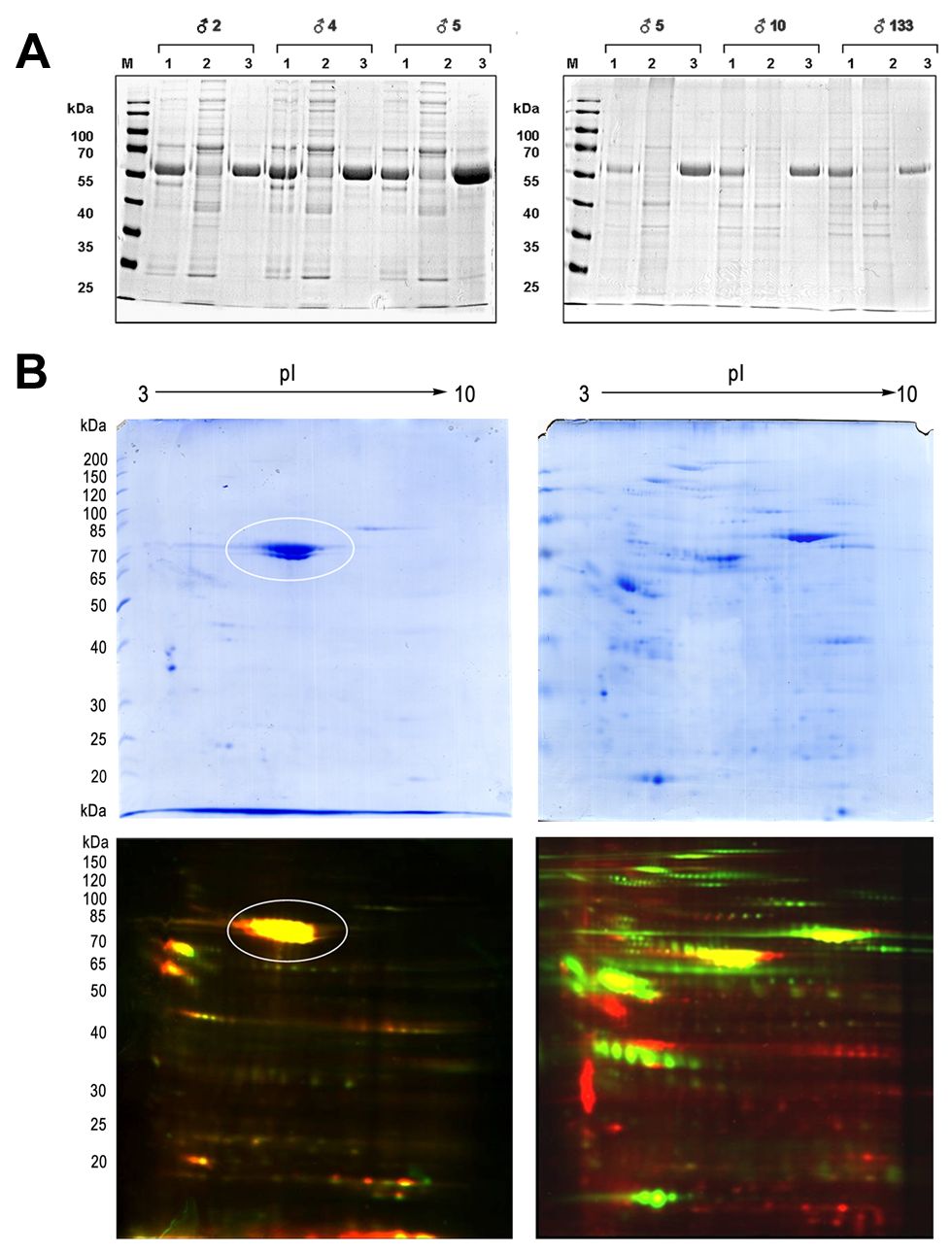

Supplement: Figure S3 — Expression profile of analysed DIGE-2D-data from ischemic HL-1 cardiomyocytes and from biopsy samples from patients with ischemic cardiomyopathy via hierarchical cluster analysis. By using an unsupervised approach comprising all spots the gels were clustered into the correct groups (n = 3). (TIF) [file pone.0031669.s003.tif]
