# Supplementary material for: DIGE Proteome Analysis Reveals Suitability of Ischemic Cardiac In Vitro Model for Studying Cellular Response to Acute Ischemia and Regeneration
Source: PLoS One. 2012 Feb 22;7(2):e31669. doi: 10.1371/journal.pone.0031669 (PMC3285183; doi:10.1371/journal.pone.0031669)
Supplement: Table S1 — Identified proteins from differentially abundant spots following ischemia in HL-1 cardiomyocytes. (DOC) [file pone.0031669.s004.doc]

| **ID^a^** | **Identification** | **Mascot Score** | | **Mw [Da]^b^** | **pI^b^** | **Sequence**  **coverage [%]** | **Matching**  **peptides** | **Accession number^c^** |
| --- | --- | --- | --- | --- | --- | --- | --- | --- |
|  |  | |  |  |  |  |  |  |
| 1 | Peroxyredoxin 3 | | 225 | 28,337 | 7.15 | 40 | 3 | P30048 |
| 2 | Tyrosin-3/tryptophan-5-monooxygenase epsilon | | 85 | 29,341 | 4.63 | 22 | 1 | P62259 |
| 3 | Succinate dehydrogenase Fp subunit | | 222 | 59,249 | 6.16 | 37 | 2 | Q8K2B3 |
| 4 | Elongation factor G | | 56 | 84,356 | 6.37 | 36 | 1 | Q8K0D5 |
| 5 | Inner membrane protein, mitochondrial, isoform CRA_b | | 77 | 86,641 | 7.02 | 20 | 1 | Q8CAQ8 |
| 6 | Vinculin | | 103 | 117,303 | 5.72 | 14 | 2 | Q64727 |
| 7 | Alpha-glycosidase 2 alpha neutral sbunit | | 413 | 109,791 | 5.75 | 31 | 2 | Q8BHN3 |
| 8 | Aconitase 2, mitochondrial | | 227 | 86,151 | 8.08 | 17 | 3 | Q99KI0 |
| 9 | Complement component 1, q subcomp. binding protein | | 214 | 31,348 | 4.77 | 37 | 2 | Q8R5L1 |
| 10 | Heat shock protein 90 - beta (HSP 84) | | 237 | 83,615 | 4.97 | 25 | 3 | P11499 |
| 11 | Heat shock protein 90 - alpha (HSP 86) | | 178 | 85,195 | 4.96 | 20 | 3 | P46633 |
| 12 | Annexin A 5 | | 254 | 35,787 | 4.83 | 38 | 3 | P48036 |
| 13 | Protein kinase C inhibitor protein 1, 14-3-3-zeta | | 111 | 27,908 | 4.70 | 28 | 1 | P61101 |
| 14 | Tyr-3/Trp-5 monooxygenase activation protein | | 55 | 20,115 | 4.51 | 27 | 4 | P62259 |
| 15 | Proliferating cell nuclear antigen | | 88 | 29,108 | 4.66 | 39 | 6 | P17918 |
| 16 | Heterogenous nuclear ribonucleoprotein L | | 85 | 60,712 | 6.65 | 11 | 5 | P14866 |
| 17 | dUTP-protein | | 51 | 21,409 | 9.63 | 87 | 5 | Q9JJ44 |
| 18 | Growth differentiation factor 6 | | 86 | 51,423 | 9.08 | 34 | 1 | P43028 |
| 19 | Aspartate aminoransferase | | 146 | 46,489 | 6.68 | 29 | 1 | P05201 |
| 20 | Electron transferring flavoprotein | | 134 | 35,360 | 8.62 | 12 | 1 | Q99LC5 |
| 21 | Heterogenous nuclear ribonucleoprotein C | | 42 | 34,421 | 4.92 | 20 | 1 | Q9Z204 |
| 22 | Vimentin | | 255 | 51,590 | 4.96 | 58 | 23 | P20152 |
| 23 | Secreted acidic cysteine rich glycoprotein | | 92 | 33,253 | 4.77 | 13 | 3 | P07214 |
| 24 | Ribosomales protein SA | | 406 | 33,008 | 4.80 | 39 | 7 | P14206 |
| 25 | Glutamate oxalacetate transaminase 2, mitochondrial | | 146 | 47,780 | 9.13 | 39 | 9 | P05202 |
| 26 | Phosphoglycerate kinase 1 | | 282 | 44,921 | 8.02 | 32 | 3 | P09411 |
| 27 | Ribonuclease/angiogenin inhibitor 1 | | 42 | 51,495 | 4.69 | 22 | 6 | Q91VI7 |
| 28 | Tubulin beta 5 | | 207 | 50,095 | 4.78 | 42 | 17 | P99024 |
| 29 | Protein phosphatase 2A | | 43 | 66,023 | 4.96 | 20 | 8 | Q76MZ3 |
| 30 | Heat shock protein 1 | | 120 | 83,571 | 4.97 | 21 | 11 | P11499 |
| 31 | Valosin containing protein | | 225 | 89,992 | 5.14 | 18 | 28 | Q01853 |
| 32 | Heat shock protein 9 | | 280 | 73,701 | 5.81 | 16 | 9 | P38647 |
| 33 | Dihydrolipoamide S-acyltransferase [precursor] | | 47 | 59,389 | 5.71 | 12 | 5 | Q8BMF4 |
| 34 | Heat schock protein 65 | | 236 | 61,074 | 5.91 | 10 | 3 | P63038 |
| 35 | put. beta-actin | | 158 | 39,446 | 5.78 | 28 | 8 | P60710 |
| 36 | Acyl Co-A thioesterase 2 | | 222 | 49,838 | 7.21 | 21 | 10 | Q9QYR9 |
| 37 | Ubiquinol-cytochrom-c reductase core protein 1 | | 298 | 53,446 | 5.81 | 10 | 3 | Q9CZ13 |
| 38 | Diacylglycerol O-acyltransferase 1 | | 36 | 57,210 | 5.46 | 4 | 1 | O75907 |
| 39 | Tu translation elongation factor, mitochondrial | | 269 | 49,876 | 7.23 | 30 | 11 | Q8BFR5 |
| 40 | Eucaryotic translation elongation factor 2 | | 251 | 96,222 | 6.41 | 21 | 16 | P58252 |
| 41 | Jak 2 Kinase | | 43 | 132,774 | 7.35 | 13 | 10 | Q62120 |
| 42 | Cyclin-dependent kinase inhibitor | | 29 | 43,280 | 9.19 | 70 | 2 | Q64364 |
| 43 | Dihydrolipoamide S-succinyltransferase | | 73 | 49,306 | 9.11 | 10 | 4 | Q9D2G2 |
| 44 | Aldehyde dehydrogenase 2, mitochondrial | | 44 | 57,015 | 7.53 | 12 | 5 | P47738 |
| 45 | Chaperonin 2 (beta) | | 133 | 57,783 | 5.97 | 21 | 9 | P80314 |
| 46 | Selenuim binding protein | | 89 | 53,051 | 5.87 | 19 | 8 | P17563 |
| 47 | Enolase 3, beta muscle | | 226 | 47,337 | 6.73 | 26 | 10 | [P13929](javascript:if(window.name=='') { window.location.href='./nil'; } else { dynPopitupType('NCBI_GENBANK__AC', 'http://www.ncbi.nlm.nih.gov/entrez/query.fcgi?db=protein&term=CAA36216'); }) |
| 48 | Pyruvate kinase M | | 201 | 58,394 | 7.58 | 21 | 9 | P52480 |
| 49 | Eukaryotic translation initiation factor 4A | | 231 | 47,095 | 6.30 | 23 | 10 | Q91VC3 |
| 50 | Ornithine aminotransferase | | 189 | 48,723 | 6.19 | 10 | 3 | P29758 |
| 51 | 3-oxoacid CoA transferase 1 | | 184 | 56,352 | 8.79 | 29 | 11 | Q9D0K2 |
| 52 | Ig heavy chain V region | | 45 | 13,939 | 6.51 | 14 | 2 | P01747 |
| 53 | Heterogenous nuclear ribonucleoprotein A2/B1 | | 581 | 36,028 | 8.67 | 49 | 14 | O88569 |
| 54 | Lactat dehydrogensase A | | 121 | 36,817 | 7.62 | 35 | 8 | P06151 |
| 55 | Phosphoglyceratmutase 1 | | 223 | 29,033 | 6.19 | 57 | 9 | Q9DBJ1 |
| 56 | Malat dehydrogensase, cytoplasm | | 91 | 36,528 | 8.83 | 40 | 9 | P14152 |
| 57 | Triosephosphate isomesase (TIM) | | 471 | 27,021 | 6.90 | 88 | 16 | P17751 |
| 58 | Nucleoside diphosphate kinase 2 | | 195 | 17,466 | 6.97 | 50 | 6 | Q01768 |
| 59 | Peptidy-prolyl-cis-trans isomerase | | 160 | 18,131 | 7.74 | 41 | 6 | P17742 |
| 60 | Oxoglutarate dehydrogenase-lipoamide | | 537 | 117,298 | 6.51 | 35 | 29 | Q91WP2 |
| 61 | Serpin h1 (Ser/Cys Proteinase Inhibitor) (HSP47) | | 446 | 46,674 | 8.90 | 39 | 10 | Q5U4D0 |
| 62 | Isovaleryl coenzym A Dehydrogenase | | 85 | 46,695 | 8.53 | 12 | 8 | Q0PGA1 |
| 63 | Nucleophosmin 1 | | 162 | 32,711 | 4.62 | 19 | 4 | Q61937 |
| 64 | Glycerinaldehyde-3-phosphate dehydrogenase | | 298 | 36,072 | 8.44 | 33 | 8 | P16858 |
| 65 | Destrin | | 126 | 18,852 | 8.41 | 18 | 2 | Q9R0P5 |
| 66 | Glycyl-t-RNA 3-phosphate dehydrogenase | | 240 | 82,597 | 6.24 | 17 | 9 | Q3TMM4 |
| 67 | Lamin B1 | | 151 | 66,842 | 5,11 | 28 | 15 | Q61791 |
| 68 | Zinc finger protein 109 | | 90 | 75,633 | 9.36 | 11 | 3 | Q61602 |
| 69 | Annexin A 6 | | 426 | 76,295 | 5.34 | 41 | 21 | Q99JX6 |
| 70 | Stress induced phosphophrotein | | 91 | 63,156 | 6.40 | 13 | 9 | Q60864 |
| 71 | Death associated protein kinase 2 | | 143 | 39,913 | 7.08 | 31 | 5 | Q9UIK4 |
| 72 | Prolin 4 hydroxylase | | 129 | 61,319 | 5.55 | 31 | 10 | Q5SX75 |
| 73 | Desmin | | 252 | 53,391 | 5.21 | 23 | 6 | P31001 |
| 74 | Reticulocalbin 3 Protein | | 237 | 36,247 | 4.67 | 47 | 8 | Q8BH97 |
| 75 | NADH-dehydrogenase (ubiquinone) 1-beta | | 145 | 21,296 | 8.19 | 38 | 4 | Q9CR21 |
| 76 | Spectrin SH-3-binding protein 1 | | 54 | 51,743 | 6.57 | 33 | 3 | Q9R1I4 |
| 77 | Fructose-bisphosphate-aldolase 1 | | 82 | 39,656 | 8.31 | 33 | 7 | P05064 |
| 78 | Granzyme K | | 40 | 29,026 | 9.95 | 14 | 2 | Q9R0K0 |
| 79 | Albumin | | 297 | 68,083 | 5.76 | 10 | 6 | Q546G4 |
| 80 | Eukaryotic translation elongation factor 4A | | 387 | 50,371 | 6.19 | 10 | 3 | P29758 |
| 81 | Chaperonin | | 236 | 61,089 | 5.67 | 10 | 3 | P63038 |

1. *Spot ID from Fig. 2b.*
2. *Data taken from NCBI database*

*c) Swiss-Prot accession.*
