# Supplementary material for: DIGE Proteome Analysis Reveals Suitability of Ischemic Cardiac In Vitro Model for Studying Cellular Response to Acute Ischemia and Regeneration
Source: PLoS One. 2012 Feb 22;7(2):e31669. doi: 10.1371/journal.pone.0031669 (PMC3285183; doi:10.1371/journal.pone.0031669)
Supplement: Table S2 — Identified proteins from spots of differentially abundant spots following reperfusion in HL-1 cardiomyocytes. (DOC) [file pone.0031669.s005.doc]

| **ID^a^** | **Identification** | **Mascot Score** | | **Mw [Da]^b^** | **pI^b^** | **Sequence**  **coverage [%]** | **Matching**  **peptides** | **Accession number^c^** |
| --- | --- | --- | --- | --- | --- | --- | --- | --- |
|  |  | |  |  |  |  |  |  |
| 82 | Peptidy-prolyl-cis-trans isomerase | | 153 | 18,131 | 7.74 | 50 | 6 | P17742 |
| 83 | Peroxyredoxin 3 | | 148 | 28,337 | 7.15 | 19 | 3 | P30048 |
| 84 | Tyrosin-3/tryptophan-5-monooxygenase epsilon | | 73 | 29,341 | 4.63 | 22 | 6 | P62259 |
| 85 | Tyrosin-3/tryptophan-5-monooxygenase epsilon | | 55 | 29,341 | 4.63 | 27 | 4 | P62259 |
| 86 | Complement component 1, q subcomp. binding protein | | 214 | 31,348 | 4.77 | 37 | 2 | Q8R5L1 |
| 87 | Heat shock protein 90 - beta (HSP 84) | | 237 | 83,615 | 4.97 | 25 | 3 | P11499 |
| 88 | Heat shock protein 90 - alpha (HSP 86) | | 178 | 85,195 | 4.96 | 20 | 3 | P46633 |
| 89 | mCG147072 [Mus musculus] | | 39 | 5,889 | 9.84 | 41 | 2 | EDL03427 |
| 90 | Heat shock protein 9 | | 81 | 73,701 | 5.81 | 16 | 8 | P38647 |
| 91 | Dihydrolipoamide S-succinyltransferase | | 73 | 49,306 | 9.11 | 10 | 4 | Q9D2G2 |
| 92 | Dihydrolipoamide S-succinyltransferase | | 71 | 49,306 | 9.11 | 10 | 4 | Q9D2G2 |
| 93 | Heat shock protein 65 | | 236 | 61,074 | 5.91 | 10 | 3 | P63038 |
| 94 | Annexin A 5 | | 244 | 35,787 | 4.83 | 38 | 1 | P48036 |
| 95 | Tu translation elongation factor, mitochondrial | | 231 | 49,876 | 7.23 | 10 | 4 | Q8BFR5 |
|  |  | |  |  |  |  |  |  |

*a) Spot ID from Fig. 2a.*

*b) Data taken from NCBI database*

*c) Swiss-Prot accession.*
