# Supplementary material for: DIGE Proteome Analysis Reveals Suitability of Ischemic Cardiac In Vitro Model for Studying Cellular Response to Acute Ischemia and Regeneration
Source: PLoS One. 2012 Feb 22;7(2):e31669. doi: 10.1371/journal.pone.0031669 (PMC3285183; doi:10.1371/journal.pone.0031669)
Supplement: Table S3 — Westernblot analysis of regulated proteins following ischemia/ischemia-reperfusion injury in HL-1 cardiomyocytes (% to histone H3). (DOC) [file pone.0031669.s006.doc]

| **Protein** |  | **8h control** | **8h ischemia** | **24h control** | **24h ischemia** |
| --- | --- | --- | --- | --- | --- |
| **PARP** | n=4 | 2.4 | 34.9 | 5.5 | 3.1 |
|  | ± SEM | 2.0 | 17.2 | 2.3 | 1.1 |
| **Rac1** | n=6 | 6.1 | 15.8 | 10.6 | 4.6 |
|  | ± SEM | 1.1 | 2.7 | 1.6 | 0.8 |
| **HSP70** | n=3 | 10.4 | 8.6 | 17.1 | 11.7 |
|  | ± SEM | 0.2 | 0.2 | 0.4 | 0.4 |
| **HSP90** | n=3 | 51.6 | 58.0 | 66.5 | 27.1 |
|  | ± SEM | 1.4 | 7.7 | 3.9 | 5.0 |
| **Connexin-43** | n=3 | 9.1 | 1.3 | 11.6 | 6.5 |
|  | ± SEM | 1.4 | 0.7 | 1.5 | 1.1 |
| **beta-Tubulin** | n=4 | 9.0 | 51.6 | 11.9 | 44.8 |
|  | ± SEM | 3.0 | 8.0 | 3.6 | 9.2 |
| **GAPDH** | n=4 | 22.8 | 68.2 | 36.5 | 80.9 |
|  | ± SEM | 3.6 | 15.5 | 7.2 | 15.7 |
| **Calsequestrin** | n=4 | 3.0 | 1.0 | 3.4 | 1.1 |
|  | ± SEM | 20.8 | 0.3 | 0.5 | 0.2 |
