# Supplementary material for: DIGE Proteome Analysis Reveals Suitability of Ischemic Cardiac In Vitro Model for Studying Cellular Response to Acute Ischemia and Regeneration
Source: PLoS One. 2012 Feb 22;7(2):e31669. doi: 10.1371/journal.pone.0031669 (PMC3285183; doi:10.1371/journal.pone.0031669)
Supplement: Table S4 — Identified proteins from differentially abundant spots following ischemic cardiomyopathy in male biopsy samples. (DOC) [file pone.0031669.s007.doc]

| **ID^a^** | **Identification of male proteinspots** | **Mascot Score** | | **Mw [Da]^b^** | **pI^b^** | **Sequence**  **coverage [%]** | **Matching**  **peptides** | **Accession number^c^** |
| --- | --- | --- | --- | --- | --- | --- | --- | --- |
|  |  | |  |  |  |  |  |  |
| 1 | Alpha-1-antichymotrypsin | | 69 | 48,834 | 5.79 | 11 | 5 | [gi|177809](http://proteo207/mascot/cgi/protein_view.pl?file=../data/20090627/F036779.dat&hit=gi|177809&px=1&ave_thresh=44&_sigthreshold=0.05&_server_mudpit_switch=0.001) |
| 2 | *^1)^*SART 3 protein | | 29 | 106,372 | 5.44 | 19 | 14 | [giI219520657](http://proteo207/mascot/cgi/protein_view.pl?file=../data/20090627/F036780.dat&hit=gi|55956899&px=1&ave_thresh=45&_sigthreshold=0.05&_server_mudpit_switch=0.001) |
| 3 | Gelsolin isoform a precursor | | 170 | 86,043 | 5.90 | 17 | 11 | gi|4504165 |
| 5 | Complement factor B | | 261 | 86,819 | 6.55 | 21 | 15 | gi|291922 |
| 6 | Chain A, C3b In Complex With A C3b Specific Fab | | 394 | 71,372 | 6.82 | 28 | 19 | [gi|224983659](http://proteo207/mascot/cgi/protein_view.pl?file=../data/20090628/F036785.dat&hit=gi|224983659&px=1&ave_thresh=44&_sigthreshold=0.05&_server_mudpit_switch=0.001) |
| 7 | Complement component 3 | | 381 | 123,428 | 6.50 | 15 | 15 | gi|194384410 |
| 8 | *^1)^*Ceruloplasmin (ferroxidase) | | 95 | 123,779 | 5.46 | 9 | 10 | [giI11999289](http://proteo207/mascot/cgi/protein_view.pl?file=../data/20090628/F036787.dat&hit=gi|177831&px=1&ave_thresh=44&_sigthreshold=0.05&_server_mudpit_switch=0.001) |
| 10 | Alpha-2-macroglobulin | | 163 | 164,600 | 6.00 | 5 | 8 | gi|112911 |
| 11 | Alpha-2-macroglobulin | | 342 | 164,600 | 6.00 | 12 | 17 | gi|112911 |
| 12 | *^1)^*Plasminogen | | 130 | 66,140 | 6.62 | 23 | 10 | giI119567978 |
| 13 | Plasminogen | | 92 | 93,233 | 7.04 | 14 | 12 | gi|190026 |
| 14 | Aconitase 2, mitochondrial precursor | | 152 | 86,113 | 7.36 | 15 | 9 | gi|4501867 |
| 15 | Aconitase 2 | | 132 | 86,113 | 7.36 | 9 | 6 | gi|4501867 |
| 16 | Complement factor H isoform a precursor | | 577 | 143,654 | 6.23 | 24 | 27 | gi|62739186 |
| 17 | Chain B, Human complement component C3 | | 261 | 114,238 | 5.55 | 22 | 21 | gi|78101268 |
| 18 | Keratin 9 | | 104 | 62,255 | 5.14 | 7 | 3 | [gi|55956899](http://proteo207/mascot/cgi/protein_view.pl?file=../data/20090628/F036798.dat&hit=gi|55956899&px=1&ave_thresh=45&_sigthreshold=0.05&_server_mudpit_switch=0.001) |
| 19 | Heat shock 70kDa protein 8 isoform 1 | | 553 | 71,082 | 5.37 | 32 | 21 | [gi|5729877](http://proteo207/mascot/cgi/protein_view.pl?file=../data/20090628/F036799.dat&hit=gi|5729877&px=1&ave_thresh=44&_sigthreshold=0.05&_server_mudpit_switch=0.001) |
| 21 | Heat shock protein 90kDa beta, member 1 | | 176 | 92,696 | 4.76 | 10 | 7 | gi|4507677 |
| 22 | *^1)^*Contactin 3 (plasmacytoma associated) | | 23 | 98,958 | 5.68 | 4 | 3 | giI119585939 |
| 23 | C9 complement protein | | 192 | 64,399 | 5.49 | 19 | 12 | gi|179726 |
| 24 | Transferrin | | 740 | 79,280 | 6.81 | 39 | 26 | gi|4557871 |
| 25 | Transferrin | | 645 | 79,280 | 6.81 | 33 | 21 | gi|4557871 |
| 26 | Keratin 1 | | 584 | 66,149 | 8.16 | 29 | 18 | [gi|7331218](http://proteo207/mascot/cgi/protein_view.pl?file=../data/20090630/F036834.dat&hit=gi|7331218&px=1&ave_thresh=44&_sigthreshold=0.05&_server_mudpit_switch=0.001) |
| 27 | Alpha-2-macroglobulin | | 273 | 71,321 | 5.47 | 20 | 11 | gi|177872 |
| 28 | Calreticulin precursor | | 59 | 48,283 | 4.29 | 3 | 2 | gi|4757900 |
| 29 | Mitochondrial ATP synthase beta | | 992 | 48,083 | 4.95 | 54 | 18 | [gi|89574029](http://proteo207/mascot/cgi/protein_view.pl?file=../data/20090630/F036841.dat&hit=gi|89574029&px=1&ave_thresh=44&_sigthreshold=0.05&_server_mudpit_switch=0.001) |
| 30 | Mitochondrial ATP synthase beta subunit precursor | | 411 | 56,525 | 5.26 | 33 | 13 | [gi|32189394](http://proteo207/mascot/cgi/protein_view.pl?file=../data/20090630/F036842.dat&hit=gi|32189394&px=1&ave_thresh=44&_sigthreshold=0.05&_server_mudpit_switch=0.001) |
| 32 | Tropomyosin 2 (beta) isoform 2 | | 77 | 33,027 | 4.63 | 16 | 4 | gi|47519616 |
| 33 | *^1)^*Tropomyosin alpha isoform 1 | | 124 | 32,746 | 4.69 | 42 | 14 | gi|14134107 |
| 34 | Annexin V chain A | | 220 | 35,840 | 4.94 | 38 | 12 | gi|809185 |
| 35 | Tyr3/trp5 -monooxygenase activation protein, epsilon | | 476 | 29,326 | 4.63 | 59 | 14 | [gi|5803225](http://proteo207/mascot/cgi/protein_view.pl?file=../data/20090630/F036853.dat&hit=gi|5803225&px=1&ave_thresh=45&_sigthreshold=0.05&_server_mudpit_switch=0.001) |
| 36 | Muscle keratin kinase | | 54 | 42,302 | 6.77 | 19 | 6 | giI21536288 |
| 39 | *^1)^*OFD 1 protein | | 36 | 27,698 | 8.90 | 27 | 4 | giI38382818 |
| 40 | Carbonic anhydrase I | | 292 | 28,909 | 6.59 | 26 | 6 | [gi|4502517](http://proteo207/mascot/cgi/protein_view.pl?file=../data/20090630/F036865.dat&hit=gi|4502517&px=1&ave_thresh=45&_sigthreshold=0.05&_server_mudpit_switch=0.001) |
| 41 | Human Igg1, kappa | | 174 | 24,324 | 6.48 | 40 | 6 | gi|1827928 |
| 42 | Triosephosphate isomerase | | 379 | 26,807 | 6.51 | 53 | 10 | gi|999892 |
| 43 | Mitochondrial dihydrolipoamide succinyltransferase | | 226 | 49,000 | 9.01 | 17 | 8 | gi|499719 |
| 44 | Mit. aldehyde dehydrogenase 2 precursor variant | | 311 | 56,845 | 6.63 | 20 | 9 | gi|62898307 |
| 45 | Cytosolic malate dehydrogenase | | 254 | 36,631 | 6.91 | 23 | 7 | gi|5174539 |
| 46 | SUCLG2 protein | | 93 | 39,085 | 5.20 | 9 | 3 | gi|73909235 |
| 47 | Creatine kinase-B | | 173 | 42,745 | 5.34 | 17 | 5 | gi|180555 |
| 48 | *^1)^*ASTN2 protein (astrotactin2) | | 31 | 42,984 | 5.46 | 13 | 3 | giI219519949 |
| 50 | Heat shock protein beta-1 (=HSP 27) | | 177 | 22,826 | 5.98 | 33 | 6 | gi|4504517 |
| 51 | Parkinson disease protein 7 | | 71 | 20,050 | 6.33 | 27 | 5 | gi|31543380 |
| 52 | Crystallin alpha B | | 124 | 20,146 | 6.76 | 509 | 6 | [gi|2852648](http://proteo207/mascot/cgi/protein_view.pl?file=../data/20090630/F036883.dat&hit=gi|2852648&px=1&ave_thresh=44&_sigthreshold=0.05&_server_mudpit_switch=0.001) |
| 53 | Crystallin, alpha B | | 112 | 22,435 | 7.18 | 25 | 5 | gi|2852648 |
| 54 | Heat shock protein beta-1(=HSP 27) | | 98 | 22,826 | 5.98 | 26 | 5 | gi|4504517 |
| 55 | Albumin complexed with myristate and azapropazone | | 292 | 68,398 | 5.57 | 28 | 16 | gi|78101694 |
| 56 | Albumin complexed with myristate and azapropazone | | 520 | 68,398 | 5.57 | 47 | 26 | gi|78101694 |
| 57 | Alpha1-antitrypsin, chain A | | 379 | 39,099 | 5.27 | 46 | 15 | gi|231240 |
| 58 | Transferrin | | 1161 | 79,280 | 6.81 | 53 | 38 | gi|4557871 |
| 59 | Chain A, crystal structure of human serum albumin | | 727 | 68,425 | 5.67 | 52 | 28 | gi|3212456 |
| 60 | Proapolipoprotein | | 223 | 28,944 | 5.45 | 60 | 15 | [gi|178775](http://proteo207/mascot/cgi/protein_view.pl?file=../data/20090701/F036891.dat&hit=gi|178775&px=1&ave_thresh=44&_sigthreshold=0.05&_server_mudpit_switch=0.001) |
|  |  | |  |  |  |  |  |  |

*a) Spot ID from Fig. 2a.*

1. *Data taken from NCBI database*

*c) NCBI accession*

*1) Data taken from MALDI-MS/MS-analysis*
