# Supplementary material for: DIGE Proteome Analysis Reveals Suitability of Ischemic Cardiac In Vitro Model for Studying Cellular Response to Acute Ischemia and Regeneration
Source: PLoS One. 2012 Feb 22;7(2):e31669. doi: 10.1371/journal.pone.0031669 (PMC3285183; doi:10.1371/journal.pone.0031669)
Supplement: Table S5 — Identified proteins from differentially abundant spots following ischemic cardiomyopathy in female biopsy samples. (DOC) [file pone.0031669.s008.doc]

| **ID^a^** | **Identification of female proteinspots** | **Mascot Score** | | **Mw [Da]^b^** | **pI^b^** | **Sequence**  **coverage [%]** | **Matching**  **peptides** | **Accession number^c^** |
| --- | --- | --- | --- | --- | --- | --- | --- | --- |
|  |  | |  |  |  |  |  |  |
| 1 | Serpin peptidase inhibitor, clade A | | 211 | 46,850 | 5.37 | 28 | 17 | gi|15990507 |
| 2 | Serine proteinase inhibitor, clade A, member 1 | | 146 | 46,878 | 5.37 | 23 | 11 | [gi|50363217](http://www.ncbi.nlm.nih.gov/blast/Blast.cgi?ALIGNMENTS=50&ALIGNMENT_VIEW=Pairwise&AUTO_FORMAT=Semiauto&CDD_SEARCH=on&CLIENT=web&COMPOSITION_BASED_STATISTICS=on&DATABASE=nr&DESCRIPTIONS=100&ENTREZ_QUERY=(none)&EXPECT=10&FILTER=L&FORMAT_BLOCK_ON_RESPAGE=None&FORMAT_OBJECT=Alignment&FORMAT_TYPE=HTML&GAPCOSTS=11+1&I_THRESH=0.001&LAYOUT=TwoWindows&MATRIX_NAME=BLOSUM62&NCBI_GI=on&PAGE=Proteins&PROGRAM=blastp&QUERY=MPSSVSWGILLLAGLCCLVPVSLAEDPQGDAAQKTDTSHHDQDHPTFNKITPNLAEFAFSLYRQLAHQSNSTNIFFSPVSIATAFAMLSLGTKADTHDEILEGLNFNLTEIPEAQIHEGFQELLRTLNQPDSQLQLTTGNGLFLSEGLKLVDKFLEDVKKLYHSEAFTVNFGDTEEAKKQINDYVEKGTQGKIVDLVKELDRDTVFALVNYIFFKGKWERPFEVKDTEEEDFHVDQVTTVKVPMMKRLGMFNIQHCKKLSSWVLLMKYLGNATAIFFLPDEGKLQHLENELTHDIITKFLENEDRRSASLHLPKLSITGTYDLKSVLGQLGITKVFSNGADLSGVTEEAPLKLSKAVHKAVLTIDEKGTEAAGAMFLEAIPMSIPPEVKFNKPFVFLMIEQNTKSPLFMGKVVNPTQK&SERVICE=plain&SET_DEFAULTS.x=9&SET_DEFAULTS.y=5&SHOW_OVERVIEW=on&WORD_SIZE=3&END_OF_HTTPGET=Yes) |
| 3 | Heat shock protein 90kDa beta, member 1 | | 426 | 92,696 | 4.76 | 26 | 18 | gi|4507677 |
| 4 | Keratin 9 | | 398 | 62,255 | 5.14 | 27 | 14 | gi|55956899 |
| 5 | Tropomyosin alpha striated muscle isoform; TPM1-alpha | | 515 | 32,747 | 4.67 | 45 | 18 | gi|49660014 |
| 7 | 80K-H protein | | 330 | 60,228 | 4.34 | 18 | 8 | gi|182855 |
| 8 | Heat shock protein HSP 90-alpha 2 | | 414 | 98,622 | 5.09 | 18 | 16 | [gi|61656603](http://proteo207/mascot/cgi/protein_view.pl?file=../data/20090625/F036710.dat&hit=gi|61656603&px=1&ave_thresh=44&_sigthreshold=0.05&_server_mudpit_switch=0.001) |
| 9 | Coagulation factor II preproprotein | | 457 | 71,475 | 5.64 | 35 | 15 | gi|4503635 |
| 10 | Heat shock 70kDa protein 5 | | 739 | 72,402 | 5.07 | 39 | 22 | gi|16507237 |
| 11 | Intermediate filament protein; | | 283 | 59,720 | 5.17 | 14 | 7 | gi|28317 |
| 12 | ^1)^Dynamin-like protein 11 | | 28 | 81,017 | 6.18 | 25 | 14 | giI5081794 |
| 13 | Heat shock 70kDa protein 8 isoform 1 | | 54 | 71,082 | 5.37 | 6 | 3 | gi|5729877 |
| 14 | heat shock 70kDa protein 8 isoform 1 | | 59 | 71,082 | 5.37 | 2 | 1 | gi|5729877 |
| 15 | Ceruloplasmin precursor | | 230 | 122,983 | 5.44 | 10 | 10 | gi|4557485 |
| 16 | Factor H | | 216 | 143,710 | 6.28 | 14 | 16 | gi|31965 |
| 17 | Alpha-2-macroglobulin | | 155 | 164,600 | 6.00 | 7 | 11 | gi|112911 |
| 18 | Macroglobulin alpha2 | | 370 | 162,072 | 5.95 | 13 | 16 | gi|224053 |
| 19 | Ceruloplasmin (ferroxidase), isoform CRA_b | | 97 | 123,779 | 5.46 | 4 | 4 | [gi|119599289](http://proteo207/mascot/cgi/protein_view.pl?file=../data/20090626/F036721.dat&hit=gi|119599289&px=1&ave_thresh=44&_sigthreshold=0.05&_server_mudpit_switch=0.001) |
| 20 | Trypsin-like serine protease | | 359 | 143,192 | 6.82 | 14 | 18 | [gi|194384367](http://proteo207/mascot/cgi/protein_view.pl?file=../data/20090626/F036723.dat&hit=gi|194384366&px=1&ave_thresh=44&_sigthreshold=0.05&_server_mudpit_switch=0.001) |
| 21 | Trypsin-like serine protease | | 359 | 143,191 | 6.82 | 14 | 18 | [gi|194384366](http://proteo207/mascot/cgi/protein_view.pl?file=../data/20090626/F036723.dat&hit=gi|194384366&px=1&ave_thresh=44&_sigthreshold=0.05&_server_mudpit_switch=0.001) |
| 22 | Complement factor B | | 221 | 86,819 | 6.55 | 15 | 12 | gi|291922 |
| 23 | Glucosidase II | | 182 | 107,289 | 5.71 | 12 | 12 | [gi|2274968](http://proteo207/mascot/cgi/protein_view.pl?file=../data/20090626/F036728.dat&hit=gi|2274968&px=1&ave_thresh=44&_sigthreshold=0.05&_server_mudpit_switch=0.001) |
| 24 | Glucosidase II | | 212 | 107,289 | 5.71 | 13 | 12 | [gi|2274968](http://proteo207/mascot/cgi/protein_view.pl?file=../data/20090626/F036729.dat&hit=gi|2274968&px=1&ave_thresh=44&_sigthreshold=0.05&_server_mudpit_switch=0.001) |
| 25 | *^1)^*Cullin 1 | | 57 | 90,306 | 8.19 | 38 | 8 | giI21358757 |
| 26 | Tropomyosin alpha striated muscle isoform; TPM1-alpha | | 660 | 32,747 | 4.67 | 60 | 15 | [gi|49660014](http://proteo207/mascot/cgi/protein_view.pl?file=../data/20090626/F036732.dat&hit=gi|49660014&px=1&ave_thresh=44&_sigthreshold=0.05&_server_mudpit_switch=0.001) |
| 27 | troponin T; TnT | | 124 | 34,555 | 5.13 | 20 | 6 | gi|408217 |
| 28 | Mit. ATP synthase, H+ transporting F1 complex beta | | 504 | 48,083 | 4.95 | 39 | 13 | [gi|89574029](http://proteo207/mascot/cgi/protein_view.pl?file=../data/20090626/F036737.dat&hit=gi|89574029&px=1&ave_thresh=44&_sigthreshold=0.05&_server_mudpit_switch=0.001) |
| 29 | Mit. ATP synthase, H+ transporting F1 complex beta | | 483 | 48,083 | 4.95 | 40 | 13 | [gi|89574029](http://proteo207/mascot/cgi/protein_view.pl?file=../data/20090626/F036737.dat&hit=gi|89574029&px=1&ave_thresh=44&_sigthreshold=0.05&_server_mudpit_switch=0.001) |
| 30 | Serum vitamin D-binding protein precursor | | 261 | 54,612 | 5.40 | 31 | 13 | gi|181482 |
| 31 | Vitamin D-binding protein | | 115 | 54,526 | 5.40 | 18 | 8 | gi|139641 |
| 32 | 2-phosphopyruvate-hydratase alpha-enolase | | 219 | 47,421 | 7.01 | 23 | 10 | gi|693933 |
| 33 | *^1)^*Rho GTPase activating protein 21 | | 29 | 16,368 | 5.85 | 23 | 3 | giI57162515 |
| 34 | *^1)^*Peptidylprolyl isomerase E (Cyclophilin E) | | 30 | 19,309 | 5.31 | 27 | 5 | giI62955333 |
| 35 | 2-phosphopyruvate-hydratase alpha-enolase | | 351 | 47,421 | 7.01 | 34 | 14 | [gi|693933](http://proteo207/mascot/cgi/protein_view.pl?file=../data/20090626/F036748.dat&hit=gi|693933&px=1&ave_thresh=44&_sigthreshold=0.05&_server_mudpit_switch=0.001) |
| 36 | Keratin 9 | | 367 | 62,255 | 5.14 | 22 | 11 | [gi|55956899](http://proteo207/mascot/cgi/protein_view.pl?file=../data/20090626/F036749.dat&hit=gi|55956899&px=1&ave_thresh=44&_sigthreshold=0.05&_server_mudpit_switch=0.001) |
| 37 | Chain A, 14-3-3 protein epsilon | | 204 | 26,912 | 4.92 | 47 | 11 | gi|67464424 |
| 38 | Tropomyosin 4 isoform 2 | | 232 | 28,619 | 4.67 | 33 | 10 | gi|4507651 |
| 39 | Tropomyosin 3 isoform 2 | | 218 | 29,243 | 4.75 | 43 | 10 | gi|24119203 |
| 40 | *^1)^*EDAR-associated death domain, isoform CRA_b | | 36 | 24,200 | 4.81 | 25 | 4 | giI119590454 |
| 41 | *^1)^*Homer 1 isoform 2 | | 30 | 25,941 | 5.38 | 42 | 8 | [guI11459943](http://proteo207/mascot/cgi/protein_view.pl?file=../data/20090627/F036754.dat&hit=gi|119611759&px=1&ave_thresh=45&_sigthreshold=0.05&_server_mudpit_switch=0.001) |
| 42 | Chain A, 14-3-3 protein theta | | 124 | 29,408 | 5.17 | 24 | 6 | gi|71042776 |
| 43 | Annexin V | | 109 | 35,840 | 4.94 | 19 | 5 | gi|809185 |
| 44 | Keratin 1 | | 612 | 66,198 | 8.16 | 35 | 19 | gi|11935049 |
| 46 | Mitochondrial heat shock 60kD protein 1 variant 1 | | 73 | 60,813 | 5.83 | 13 | 7 | gi|189502784 |
| 47 | Chain A, TapasinERP57 heterodimer | | 157 | 54,541 | 5.61 | 22 | 9 | gi|220702506 |
| 48 | Apolipoprotein A-IV precursor | | 310 | 45,353 | 5.33 | 36 | 14 | gi|178757 |
| 49 | Beta actin variant | | 216 | 39,543 | 5.4 | 32 | 12 | [gi|194385944](http://proteo207/mascot/cgi/protein_view.pl?file=../data/20090627/F036764.dat&hit=gi|194385944&px=1&ave_thresh=44&_sigthreshold=0.05&_server_mudpit_switch=0.001) |
| 50 | ^1)^DEAD box helicases | | 37 | 71,710 | 6.18 | 15 | 8 | gI194388152 |
| 51 | Apolipoprotein E | | 58 | 36,302 | 5.65 | 3 | 1 | gi|178849 |
| 52 | Tropomyosin 2 (beta) isoform 2 | | 431 | 33,027 | 4.63 | 52 | 13 | gi|47519616 |
| 53 | Tropomyosin 1 alpha chain isoform 2 | | 324 | 32,715 | 4.70 | 40 | 12 | gi|63252902 |
| 54 | Tropomyosin alpha striated muscle isoform; TPM1-alpha | | 440 | 32,747 | 4.67 | 47 | 12 | gi|49660014 |
| 56 | *^1)^*Inhibitor of apoptosis protein1 | | 25 | 31,279 | 5.17 | 24 | 4 | giI110590599 |
| 57 | *^1)^*Adaptor-related protein complex 3, mu 2 subunit | | 97 | 76,988 | 6.85 | 32 | 44 | giI197209857 |
| 58 | Apoptosis-inducing factor (AIF) | | 60 | 40,615 | 9.16 | 2 | 1 | gi|14318424 |
| 59 | Transferrin | | 632 | 79,280 | 6.81 | 34 | 21 | gi|4557871 |
|  |  | |  |  |  |  |  |  |

*a) Spot ID from Fig. 2a.*

1. *Data taken from NCBI database*

*c) NCBI accession*

*1) Data taken from MALDI-MS/MS-analysis*
